# Glycolysis‐Histone Lactylation Crosstalk Drives TXNIP‐NLRP3‐Mediated PANoptosome Assembly and PANoptosis Activation Underlying Diabetic Retinopathy Pathogenesis

**DOI:** 10.1002/mco2.70351

**Published:** 2025-09-14

**Authors:** Xiaoting Xi, Qianbo Chen, Jia Ma, Xuewei Wang, Yuxin Zhang, Qiuxia Xiong, Xiaolei Liu, Yuan Xia, Yan Li

**Affiliations:** ^1^ Ophthalmology Department The First Affiliated Hospital of Kunming Medical University Kunming City China; ^2^ Yunnan Key Laboratory of Laboratory Medicine The First Affiliated Hospital of Kunming Medical University Kunming City China; ^3^ Neurology Department The First Affiliated Hospital of Kunming Medical University Kunming City China

**Keywords:** diabetic retinopathy, glycolysis, histone lactylation, PANoptosis, thioredoxin interacting protein

## Abstract

Diabetic retinopathy (DR), a major cause of vision loss in adults, involves aberrant metabolism and inflammation. This study investigated the interplay between glycolysis, histone lactylation, and PANoptosis in DR using human retinal pigment epithelial (RPE) cells under high glucose and diabetic mouse models. Results demonstrated a positive feedback loop where enhanced glycolysis increased histone lactylation, which in turn further promoted glycolysis. This cycle activated the expression of thioredoxin interacting protein (TXNIP) and NOD‐like receptor thermal protein domain associated protein 3 (NLRP3), leading to PANoptosome formation and triggering PANoptosis, a coordinated cell death pathway contributing to DR pathology. Crucially, experiments manipulating TXNIP expression (via RNAi or overexpression) confirmed its central role in linking histone lactylation to NLRP3 activation and PANoptosome assembly. Importantly, inhibiting glycolysis or downregulating TXNIP successfully reduced histone lactylation, suppressed PANoptosome formation, and alleviated PANoptosis. These findings establish that the glycolysis‐histone lactylation axis, mediated by TXNIP/NLRP3 signaling, drives PANoptosis in RPE cells through PANoptosome formation, playing a critical role in DR development. Targeting this specific pathway presents a promising new therapeutic strategy for diabetic retinopathy.

## Graphical abstract

This study illustrates how high glucose triggers glycolysis, ROS production, and lactic acid accumulation, leading to TXNIP upregulation and PANOptosome formation. These complexes activate apoptosis (cleaved caspase‐3), pyroptosis (GSDME), and necroptosis (MLKL), contributing to diabetic retinopathy. The diagram highlights the role of EP300 and ERG in TXNIP promoter regulation, offering insights into potential therapeutic targets.

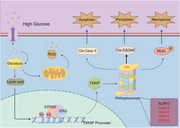

## 1 Introduction

Diabetic retinopathy (DR) is the most common complication of diabetes and is also one of the leading causes of blindness globally [1]. Multiple molecular mechanisms have been identified in DR, such as the formation of advanced glycation end products, oxidative stress, polyol accumulation, and activation of protein kinase C [2]. These mechanisms act together to cause mitochondrial membrane potential (MMP) imbalance, cytochrome c (CYCS) release, and a large amount of reactive oxygen species (ROS) production. These changes affect cell metabolism and signal transduction, further leading to overexpression of inflammatory cytokines and vascular endothelial growth factor (VEGF). VEGF has been recognized as an important target for the treatment of DR and macular edema [3]. However, the exact pathogenesis of DR remains unclear, so it is of great significance to further explore the pathophysiological mechanisms of DR for the prevention and treatment of this destructive disease.

In DR, numerous retinal cell types are impacted, including severe neuronal loss in the retina [4], Muller cell malfunction [5], and the activation of astrocytes and microglia [6], which have been extensively examined. These findings significantly enhance our comprehension of the multiple signaling pathways and the numerous cell types within the retina that play a pivotal role in the development of DR. Nevertheless, the role of the retinal pigment epithelium (RPE) during the early stages of DR remains unclear when compared to our understanding of the function of neurosensory retinal cells. The RPE is a single layer composed of polarized, multifunctional pigment cells that form the outer blood‐retinal barrier (BRB) and are crucial for maintaining retinal function [7]. Recent studies have suggested that the RPE may also contribute to the development of DR [8]. The loss of insulin receptor‐mediated signal transduction within the RPE reduces the levels of ROS and pro‐inflammatory cytokine expression in the retina of diabetic mice, providing evidence that the RPE may be involved in the development of DR [9]. Nevertheless, the mechanisms underlying diabetes‐induced changes in the RPE remain unclear.

PANoptosis, a phenomenon where apoptosis, necrosis, and necroptosis occur simultaneously and can be simultaneously regulated, has been observed in the pathophysiological processes of certain diseases [10]. Numerous studies have reported on the regulation of PANoptosis by the PANoptosome complex, which is assembled by key regulators of apoptosis, necrosis, and necroptosis [11]. This protein complex simultaneously regulates apoptosis, necrosis, and necroptosis [12]. PANoptosis has been associated with the development of various systemic diseases in humans, including infectious diseases, cancers, neurodegenerative disorders, and inflammatory diseases [13, 14, 15]. These findings suggest that PANoptosis may play a larger role in DR. However, there is currently a lack of literature reporting on this topic. Therefore, we aim to explore the potential regulatory mechanisms of PANoptosis in DR and further clarify the pathophysiological mechanisms of DR.

Prolonged exposure to a high‐sugar environment disrupts cellular respiration, leading to increased reliance on anaerobic glycolysis as the primary energy‐producing pathway. This metabolic shift not only compensates for damage to the respiratory enzyme system but also significantly elevates lactate production [16]. Glycolysis is a central process in glucose metabolism and becomes more active under various cellular stimuli, making it a dominant energy source in many pathological conditions [17]. The accumulation of lactate under such conditions can trigger histone lysine lactoacylation—a newly identified epigenetic modification [18]. In recent years, histone lactylation—specifically histone H3 lysine 18 lactylation (H3K18la)—has been reported to regulate various biological processes, such as tumorigenesis [19], tumor immune escape [20], and the metabolic reprogramming of cancer cells [21]. However, the regulatory mechanism of histone lactate in DR remains elusive.

In this study, we demonstrate that the TXNIP/NLRP3 signaling pathway serves as a critical link between histone lactylation and PANoptosis in retinal pigment epithelial (RPE) cells, thereby contributing to the progression of DR. Prolonged exposure to a high‐glucose environment leads to metabolic dysregulation characterized by enhanced glycolysis in RPE cells, resulting in excessive lactate accumulation. This increased lactate promotes histone lactylation, a newly identified epigenetic modification, particularly in the promoter region of TXNIP. Enhanced histone lactylation upregulates TXNIP expression, which in turn activates the NLRP3 inflammasome. Subsequently, NLRP3 initiates the formation of the PANoptosome, triggering PANoptosis—a convergent pathway involving pyroptosis, apoptosis, and necroptosis—leading to retinal cell death and inflammation. Our findings provide novel insights into the molecular mechanisms underlying DR and highlight the pivotal roles of metabolic reprogramming, histone lactylation, and PANoptosis in the pathogenesis of this sight‐threatening disease. These results may offer new therapeutic targets for the prevention and treatment of DR.

## 2 Result

### 2.1 RPE Plays a Vital Role in the Pathogenesis of DR

**FIGURE 1:**
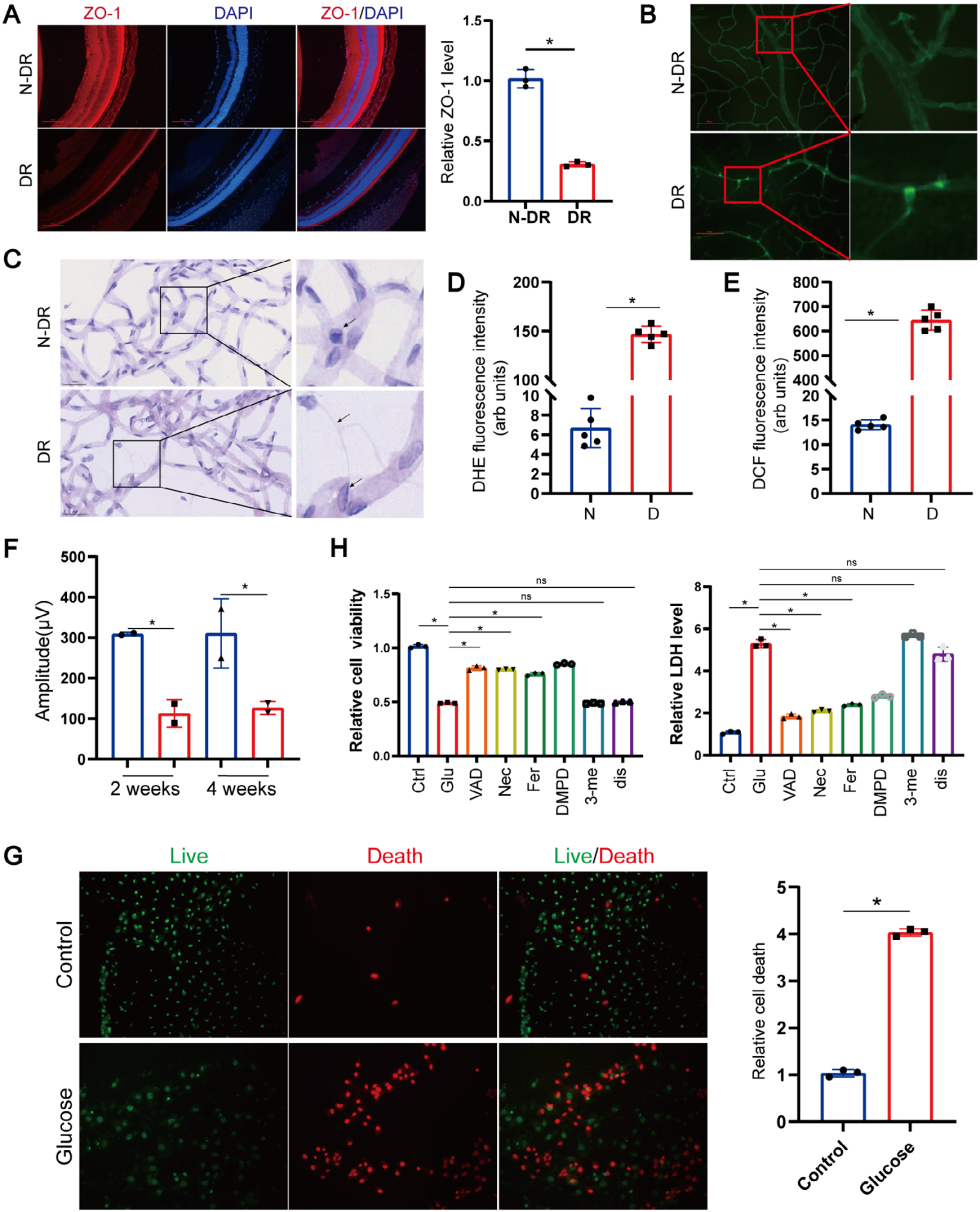
Massive RPE death impacts the progression of diabetic retinopathy (DR). (A) Representative images of immunofluorescence (IF) staining in retinal sections from diabetic and control mice (*n* = 3). Nuclei were counterstained with DAPI. Scale bar: 100 µm. (B) Representative images illustrating leukostasis in retinal vasculature, assessed by FITC‐conjugated concanavalin A perfusion labeling followed by flat‐mount staining. Scale bar: 100 µm. (C) Representative micrographs of retinal vasculature visualized using periodic acid–Schiff (PAS) staining in diabetic and age‐matched non‐diabetic mice. Arrows indicate acellular capillaries. Scale bar: 25 µm. (D and E) Quantification of dihydroethidium (DHE) fluorescence intensity (D) and dichlorofluorescein (DCF) fluorescence (E) in retinal sections as indicators of superoxide and reactive oxygen species (ROS) levels, respectively. Data represent mean ± SD from five biological replicates (*n* = 5). Statistical significance was determined by one‐way ANOVA with Bonferroni correction. *p < 0.05 versus control. (F) Quantification of electroretinography (ERG) a‐ and b‐wave amplitudes under scotopic conditions to assess retinal function. Data are presented as mean ± SD (*n* = 3). One‐way ANOVA with Bonferroni post hoc test was used. *p < 0.05. (G) Live/dead viability/cytotoxicity assay performed using calcein AM (green fluorescence for live cells) and EthD‐III (red fluorescence for dead cells) (*n* = 3). Scale bar: 100 µm. (H) Assessment of cell viability (left) and cytotoxicity (right) following 48‐h treatment of ARPE‐19 cells with 25 mM high glucose, in combination with apoptosis inhibitor Z‐VAD‐FMK (VAD, 25 µM), necroptosis inhibitor necrostatin (Nec, 20 µM), ferroptosis inhibitor ferrostatin‐1 (Fer, 10 µM), pyroptosis inhibitors Ac‐DMPD‐CMK/DMLD‐CMK (DMPD/DMLD, 20 µM), disulfiram (Dis, 1 µM), or autophagy inhibitor 3‐methyladenine (3‐Me, 10 µM). Data represent mean ± SD from three independent experiments (*n* = 3). Statistical analysis was performed using one‐way ANOVA with Bonferroni correction. *p < 0.05 versus control.

DR, a leading cause of blindness globally, involves critical functions of RPE cells in its pathological mechanisms. To investigate the pivotal role of RPE in DR pathogenesis, a mouse model of DR was established through STZ treatment. Immunofluorescence results revealed reduced RPE cells in the retinas of DR mice (Figure 1A). Given that ICAM‐1 mediates leukocyte attachment to retinal endothelial cells, promoting leukostasis—a key pathogenic event in DR progression [22]—we validated the DR mouse model via leukostasis and acellular capillary assays. Results indicated increased leukostasis in the diabetic mouse retinal vasculature (Figure 1B) and a rise in acellular capillaries with pericyte loss (Figure 1C). Diabetes‐induced elevation of retinal superoxide and ROS is reported, highlighting oxidative stress as a pivotal factor in diabetic retinal microvascular damage [23, 24]. Thus, we assessed retinal ROS generation in these mouse models using freshly isolated retinas incubated with fluorophore (reacting with superoxide) and retinal cryosections stained with dihydroethidium (DHE, reacting with superoxide) and 2',7'‐dichlorodihydrofluorescein diacetate (DCF, reacting with ROS). We observed a significant increase in diabetes‐induced superoxide and ROS levels (Figure 1D,E). Retinal degenerative changes are reported as early events in DR development [25, 26]; accordingly, we tested retinal function via electroretinography (ERG) (Figure 1F; Figure S1A), indicating severe impairment of both retinal and RPE cells. This further emphasizes the crucial role of RPE in DR pathogenesis. Exploring the mechanism behind RPE impairment, we found massive RPE death under high‐glucose conditions using the LIVE/DEAD Assay Kit (Figure 1G). To identify the type of cell death occurring, inhibitors of common cell death pathways [27, 28], including apoptosis, necroptosis, ferroptosis, pyroptosis, and autophagy, were used to rescue high‐glucose‐induced cell death. Remarkably, apoptosis, necroptosis, ferroptosis, and pyroptosis inhibitors reversed the decrease in cell viability and increase in cytotoxicity induced by high glucose, whereas the autophagy inhibitor did not (Figure 1H). However, none of the inhibitors fully restored levels observed in controls (Figure 1H), suggesting that high glucose concurrently triggers multiple forms of cell death (PANoptosis) in RPE.

### 2.2 RPE Cells Undergo PANoptosis With Interconnected Apoptosis, Pyroptosis, and Necroptosis

**FIGURE 2:**
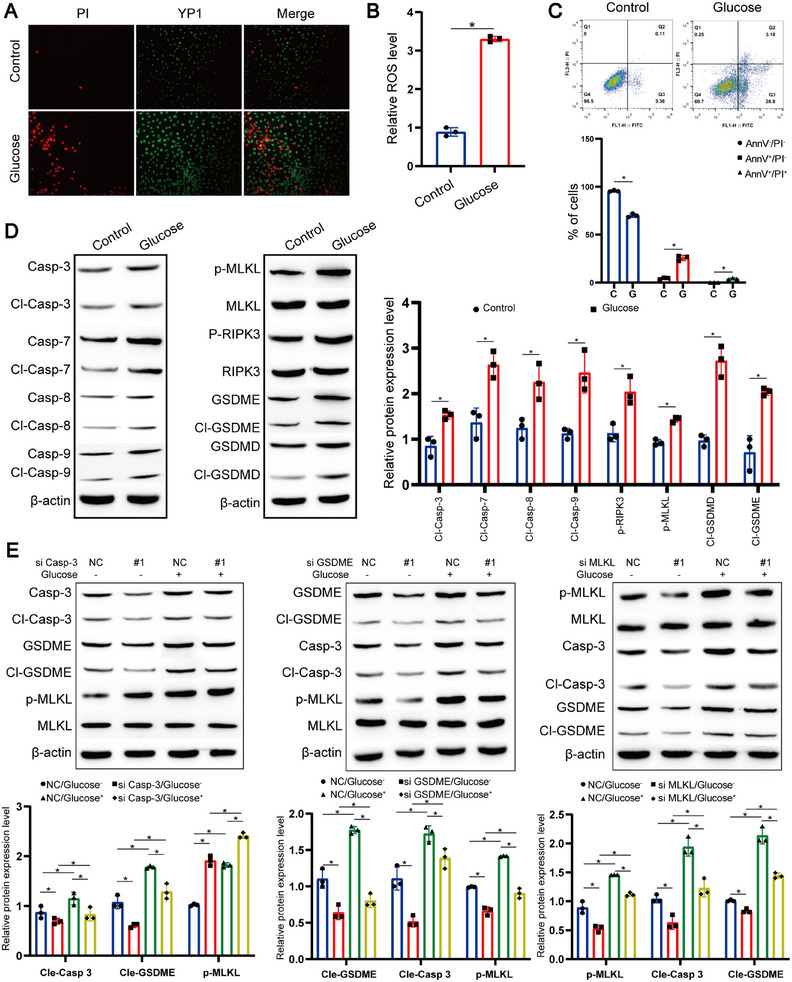
ARPE‐19 cells undergo PANoptosis with interconnected apoptosis, pyroptosis, and necroptosis. (A) Representative images of YP1‐positive (green, DNA damage indicator) and propidium iodide (PI)‐positive (red, membrane disruption) staining in ARPE‐19 cells after high glucose treatment. Nuclei were counterstained with DAPI. Scale bar = 100 µm. (B) Detection and quantification of reactive oxygen species (ROS) levels using DCFDA fluorescent probe. Data represent mean ± SD from three biological replicates (*n* = 3). One‐way ANOVA with Bonferroni correction was applied. *p < 0.05. (C) Flow cytometry analysis and quantification of annexin V/PI‐stained cells to evaluate apoptosis and cell death. Data are shown as mean ± SD (*n* = 3). Statistical significance was calculated using one‐way ANOVA with Bonferroni post hoc test. *p < 0.05 versus control. (D) Representative western blot images showing expression and cleavage status of caspase‐3, ‐7, ‐8, ‐9; phosphorylated and total MLKL; phosphorylated and total RIP3; GSDME and cleaved GSDME; GSDMD and cleaved GSDMD in ARPE‐19 cells after 48 h of high glucose treatment. β‐Actin served as loading control. Data represent mean ± SD from three independent experiments (*n* = 3). Statistical significance was assessed by one‐way ANOVA with Bonferroni correction. *p < 0.05. (E) Western blot analysis of phosphorylated MLKL, total MLKL, caspase‐3, cleaved caspase‐3, GSDME, and cleaved GSDME expression in ARPE‐19 cells transfected with siRNA targeting caspase‐3, GSDME, or MLKL. Protein levels were normalized to β‐actin. Data represent mean ± SD from three independent experiments (*n* = 3). Statistical significance was assessed by one‐way ANOVA with Bonferroni correction. *p < 0.05.

To further substantiate the occurrence of PANoptosis, we conducted in vitro cellular investigations. We observed a significant increase in the number of dead cells following high‐glucose treatment (Figure 2A; Figure S2A). In line with the in vivo findings (Figure 1E), we also detected increased ROS levels in vitro (Figure 2B; Figure S2B). Moreover, high glucose administration led to a higher count of early/late apoptotic cells and a substantial reduction in viable cell numbers (Figure 2C; Figure S2C). Further, Western blot analyses revealed significantly elevated expressions of cleaved‐caspase‐3, p‐MLKL, and cleaved‐GSDME, markers associated with apoptosis, necroptosis, and pyroptosis, respectively (Figure 2D; Figure S2D). Collectively, these data indicate the activation of PANoptosis in RPE cells under high‐glucose conditions.

Research has shown that various cell death programs operate in alternation and exhibit extensive crosstalk, rendering cells resistant to one pathway yet sensitive to death through another under certain circumstances [29, 30]. Therefore, we blocked one component of PANoptosis to explore its impact on other components and found that silencing caspase‐3 in RPE cells significantly augmented MLKL phosphorylation but reduced GSDME cleavage. Conversely, silencing GSDME decreased caspase‐3 cleavage and MLKL phosphorylation, while MLKL knockdown diminished cleavage of both caspase‐3 and GSDME (Figure 2E; Figure S2E).

These findings establish that RPE cells undergo PANoptosis, encompassing apoptosis, necroptosis, and pyroptosis, under high‐glucose environments. This prompts us to speculate about the presence of a defined signaling pathway governing this process.

### 2.3 TXNIP/NLRP3 Signaling Activation Promotes PANoptosis in Cells

**FIGURE 3:**
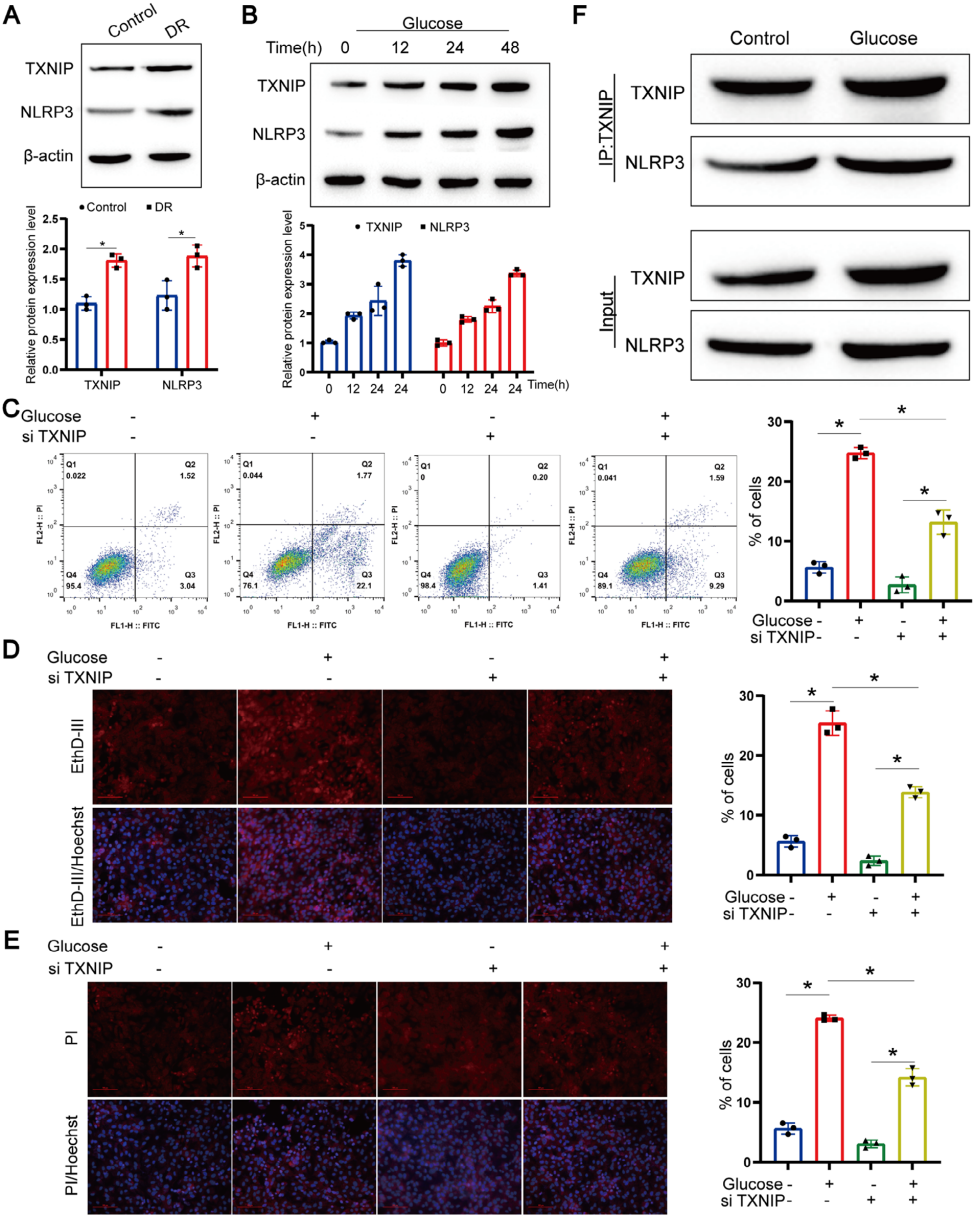
Activation of TXNIP/NLRP3 signaling promotes PANoptosis in cells. (A) Representative Western blot images of TXNIP and NLRP3 in retinal tissues from diabetic and control mice. β‐Actin was used as loading control. Data represent mean ± SD from three independent experiments (*n* = 3). Statistical significance was assessed by one‐way ANOVA with Bonferroni correction. *p < 0.05. (B) Representative western blot images of TXNIP and NLRP3 expression in ARPE‐19 cells treated with high glucose. Data represent mean ± SD from three independent experiments (*n* = 3). Statistical significance was assessed by one‐way ANOVA with Bonferroni correction. *p < 0.05. Representative images depicting TUNEL‐positive apoptotic cells (C), EthD‐III‐positive pyroptotic cells (D), and PI‐positive necroptotic cells (E) in ARPE‐19 cells treated with high glucose alone or in combination with siTXNIP. Nuclei were stained with DAPI. Scale bar = 100 µm. Data represent mean ± SD from three independent experiments (*n* = 3). Statistical significance was assessed by one‐way ANOVA with Bonferroni correction. *p < 0.05. (F) Co‐immunoprecipitation (Co‐IP) analysis showing interaction between TXNIP and NLRP3 in ARPE‐19 cells treated with high glucose. Whole‐cell lysates were immunoprecipitated with anti‐TXNIP antibody and analyzed by Western blotting with anti‐NLRP3. IgG was used as negative control. Densitometric quantification of co‐IP band intensity is shown.

As reported in literature, NLRP3, AIM2, NLRC4, and Pyrin, along with ASC, caspase‐1, caspase‐8, and RIPK3, are identified as components of a large multiprotein complex driving PANoptosis [31]. Studies have demonstrated that mitochondrial ROS‐TXNIP/NLRP3 axis activation contributes to tubular injury in diabetic nephropathy patients [32]. Consequently, we examined the expression of TXNIP/NLRP3 in retinal tissues of our model mice, observing an elevation compared to controls (Figure 3A); similar outcomes were obtained from in vitro cell models (Figure 3B; Figure S3A).

To further probe the impact of the TXNIP/NLRP3 signaling pathway on PANoptosis, we knocked out or overexpressed TXNIP (Figure S3B,C). Initial morphological assessments revealed that high glucose treatment significantly induced features of panoptosis, including apoptosis (Figure 3C), pyroptosis (Figure 3D), and necroptosis (Figure 3E) in RPE cells. Knockout of TXNIP mitigated these forms of cell death (Figures 3C–E), whereas TXNIP overexpression exacerbated PANoptosis (Figure S3D–F).

Delving deeper into the molecular mechanisms involving TXNIP/NLRP3, co‐immunoprecipitation (Co‐IP) analysis disclosed a robust interaction between NLRP3 and TXNIP under high glucose conditions (Figure 3F), which was further confirmed by pull‐down experiments demonstrating enhanced NLRP3‐TXNIP interaction upon high glucose exposure (Figure S3G).

Collectively, these findings suggest that high glucose environments activate TXNIP/NLRP3 signaling in RPE cells, leading to PANoptosis. Additionally, flow cytometric analysis revealed increased oxidative stress and ROS levels in RPE cells subjected to high glucose (Figure S3H), implying aberrant glycolysis in RPE cells under hyperglycemic conditions.

### 2.4 NLRP3 Interacts With NLRP12 and Cell Death Molecules to Drive PANoptosis

**FIGURE 4:**
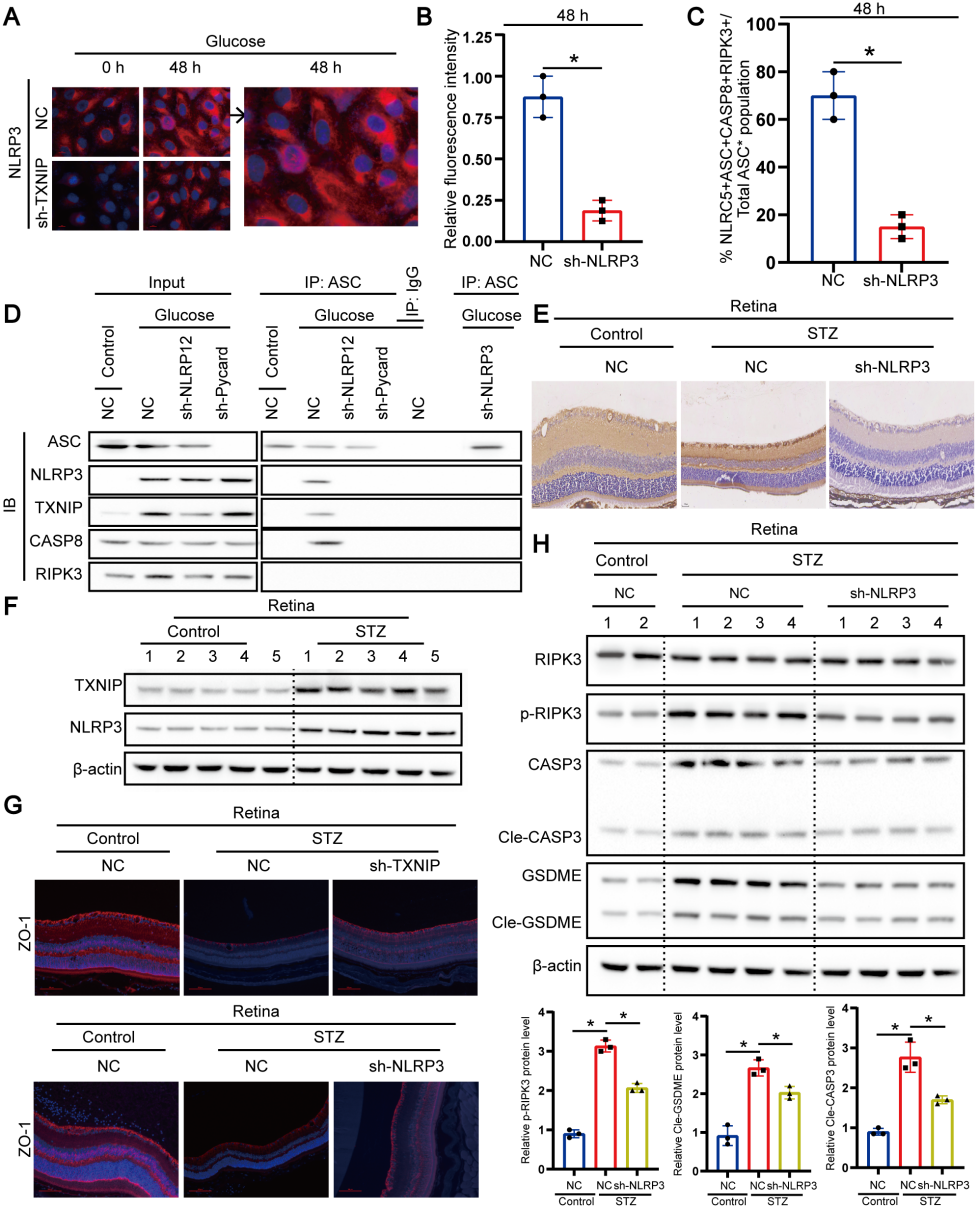
NLRP3 interacts with NLRP12 and cell death molecules to drive PANoptosis. (A and B) NC and Nlrp3 knockout ARPE‐19 cells were either left unstimulated (0 h) or treated with high glucose for 48 h and immunostained for NLRP3. Nuclei were counterstained with DAPI. A broader field of view for NC cells at 48 h is shown (A), and quantification of NLRP3‐positive aggregates is provided (B). Scale bar = 10 µm. Data represent mean ± SD (*n* = 3). One‐way ANOVA with Bonferroni post hoc test was used. *p < 0.05. (C) NC and Nlrp3 knockout ARPE‐19 cells were treated with high glucose for 48 h and co‐stained for NLRP3, ASC, caspase‐8 (CASP8), and RIPK3. Representative images of cells containing co‐localized puncta are shown. Enlarged views of selected regions are displayed on the right. Scale bar = 10 µm. (D) Immunoblot (IB) analysis of ASC, TXNIP, NLRP3, CASP8, and RIPK3 following immunoprecipitation (IP) with IgG control or anti‐ASC antibodies in NC and Nlrp3 knockout ARPE‐19 cells after high glucose treatment. Densitometric quantification is shown. (E) Representative images showing immunohistochemistry (IHC)‐stained retinal sections from wild‐type (WT) and Nlrp3 knockout mice 2 weeks after intraperitoneal injection of PBS or streptozotocin (STZ). Staining for cleaved caspase‐3, GSDMD‐N, and phosphorylated MLKL was performed. Scale bar = 100 µm. (F) Western blot analysis of NLRP3 expression in retinal tissues from WT mice treated with PBS or STZ for 2 weeks. β‐Actin served as loading control. Data represent mean ± SD from five biological replicates (*n* = 5). Statistical significance was assessed by one‐way ANOVA with Bonferroni correction. *p < 0.05 versus control. (G) Representative images of ZO‐1 immunofluorescence staining to assess retinal vascular integrity in WT and Nlrp3 knockout mice following PBS or STZ treatment. Scale bar = 100 µm. (H) Western blot analysis of RIPK3, CASP3, and GSDME expression levels in retinal tissues from WT and Nlrp3 knockout mice at 2 weeks post‐PBS or STZ injection. β‐Actin served as internal control. Data represent mean ± SD from three biological replicates (*n* = 3). Statistical analysis was conducted using one‐way ANOVA with Bonferroni post hoc test. *p < 0.05 versus control.

To further investigate the regulation of PANoptosis by *Nlrp3*, we next examined whether *Nlrp3* influenced the formation of the PANoptosome in response to high glucose treatment. Through immunofluorescence (Figure 4A,B), we observed increased expression of *Nlrp3* under high glucose conditions. We also observed co‐localization of the key PANoptosome molecules—apoptosis‐associated speck‐like protein containing a caspase activation and recruitment domain (ASC), caspase‐8, and receptor‐interacting protein kinase 3 (*Ripk3*)—with *Nlrp3* in RPE cells after high glucose treatment (Figure 4C), indicating that *Nlrp3* is essential for the formation of the PANoptosome complex. Previous studies have shown that NLRP12 interacts with ASC, caspase‐8, and RIPK3 to drive PANoptosis. To determine the role of NLRP3 in this complex, we performed endogenous immunoprecipitation in NC, Nlrp12 knockout, Pycard knockout, and Nlrp3 knockout RPE cells following high glucose treatment. We observed the formation of a multiprotein complex containing ASC, RIPK3, caspase‐8, and NLRP3 in NC cells, and the formation of this complex was abolished in the absence of NLRP12, ASC, or NLRP3 (Figure 4D). These results suggest that NLRP3, like NLRP12 and ASC, is a critical component of the PANoptosome complex that drives PANoptosis in response to high glucose stimulation. Collectively, these results indicate that *Nlrp3* forms a PANoptosome complex with *Nlrp12* to drive high glucose‐mediated inflammatory cell death. To determine whether *Txnip/Nlrp3* mediates disease pathogenesis, we utilized an established model of DR, in which mice were treated with STZ. Early studies have shown that *Nlrp3* is involved in driving inflammation and renal damage in diabetes models. Therefore, IHC staining revealed more severe retinal tissue damage in NC mice following STZ treatment compared to *Nlrp3* knockout mice (Figure 4E). Compared with the control treatment group, *Nlrp3* expression in the retinal tissues of NC mice was significantly increased after STZ treatment (Figure 4F). We observed a decrease in ZO‐1 staining in the retinal tissues of NC mice after STZ treatment, compared with the control treatment group (Figure 4G). Moreover, ZO‐1 expression in the retinal tissues of *Nlrp3* knockout mice was significantly enhanced compared to NC mice (Figure 4G). Consistently, we also observed a reduction in the activation of *Ripk3*, *Caspase‐3*, and *Gsdme* in the retinal tissues of *Nlrp3* knockout mice after STZ treatment (Figure 4H), indicating that *Txnip/Nlrp3*‐mediated cell death plays a critical role in STZ‐induced DR damage. Collectively, these results suggest that *Txnip/Nlrp3*‐mediated cell death plays a key role in DR.

### 2.5 Dysregulated Glycolysis Elevates Histone Lactylation in RPE Cells, Promoting PANoptosis

**FIGURE 5:**
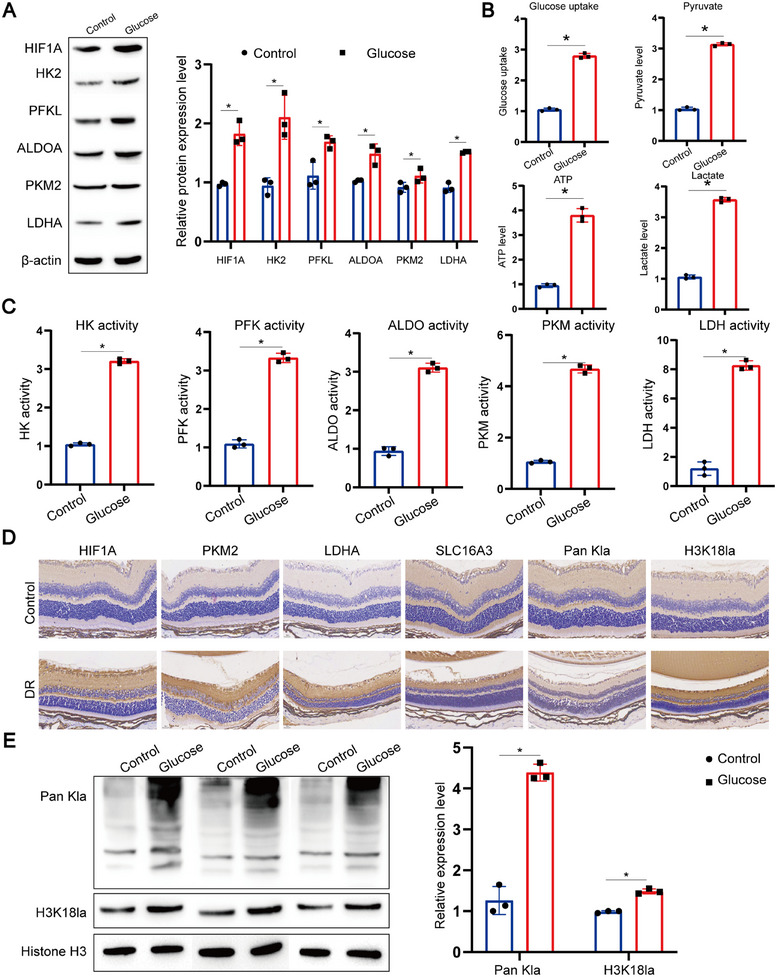
Aberrant glycolysis elevates histone lactylation in RPE cells. (A) Representative western blot images showing changes in HIF1A and glycolytic pathway proteins (HK2, PFKL, ALDOA, PKM2, LDHA) in murine RPE (mRPE) cells after high glucose treatment. β‐Actin was used as loading control. Data represent mean ± SD from three biological replicates (*n* = 3). One‐way ANOVA with Bonferroni correction was applied. *p < 0.05 versus control. (B and C) Quantitative detection of glucose uptake, pyruvate levels, lactate production, ATP concentration, and enzymatic activities (hexokinase [HK], phosphofructokinase [PFK], aldolase [ALDO], pyruvate kinase [PKM], lactate dehydrogenase [LDH]) in ARPE‐19 cells using commercial assay kits. Data represent mean ± SD from three biological replicates (*n* = 3). One‐way ANOVA with Bonferroni correction was applied. *p < 0.05 versus control. (D) Representative immunohistochemistry (IHC) images detecting HIF1A, PKM2, LDHA, SLC16A3 (MCT4), and histone lactylation (H3K18la) levels in retinal tissues from diabetic and control mice. Scale bar: 100 µm. (E) Representative western blot images assessing global histone lactylation levels in ARPE‐19 cells after exposure to high glucose. Histones were extracted and probed with anti‐H3K18la antibody. Data represent mean ± SD from three biological replicates (*n* = 3). Statistical analysis was conducted using one‐way ANOVA with Bonferroni post hoc test. *p < 0.05 versus control.

Chronic hyperglycemia disrupts mitochondrial balance, generating copious ROS radicals and causing endothelial injury, which in turn facilitates local hypoxia [33]. Under hypoxic conditions, most eukaryotic cells can shift their primary metabolic strategy from mitochondrial respiration to enhanced glycolysis to maintain ATP levels, with prolonged hypoxia leading to abnormal intracellular glycolytic activity [34]. We assayed the expression of HIF1A and various glycolytic proteins by Western Blot, confirming aberrant glycolysis in RPE cells under high glucose conditions (Figure 5A; Figure S4A). This was accompanied by heightened glucose uptake, pyruvate levels, lactate production, and ATP concentrations in RPE cells (Figure 5B; Figure S4B). Enzymatic activities during glycolysis were measured using dedicated kits, revealing a general increase (Figure 5C; Figure S4C).

Immunohistochemistry (IHC) of mouse model tissues further validated the glycolytic anomaly by detecting *Hif1a* and several glycolytic proteins (Figure 5D; Figure S4D), alongside a marked increase in total histone lactylation and specifically H3K18la levels. Western Blot analyses concurred with these observations (Figure 5E; Figure S4E).

In vitro experiments were then conducted to further substantiate the impact of glycolysis on histone lactylation. Western Blot results showed that with extended glucose exposure, there was a rise in total histone lactylation and H3K18la levels in RPE cells (Figure S5A). Treatment with glycolysis inhibitors (2DG and Oxamate) led to a dose‐dependent decrease in total histone lactylation and H3K18la (Figure S5B). Correspondingly, upon treatment with these inhibitors, western blot analysis displayed a reduction in the expression of proteins associated with PANoptosis (apoptosis, pyroptosis, and necroptosis) as inhibitor concentration increased (Figure S5C).

These findings highlight the regulatory role of glycolysis in PANoptosis of RPE cells. Consequently, we posit that glycolysis, through histone lactylation at the TXNIP promoter region, modulates PANoptosis in RPE cells.

### 2.6 Histone Lactylation Activates TXNIP Transcription in RPE Cells

**FIGURE 6:**
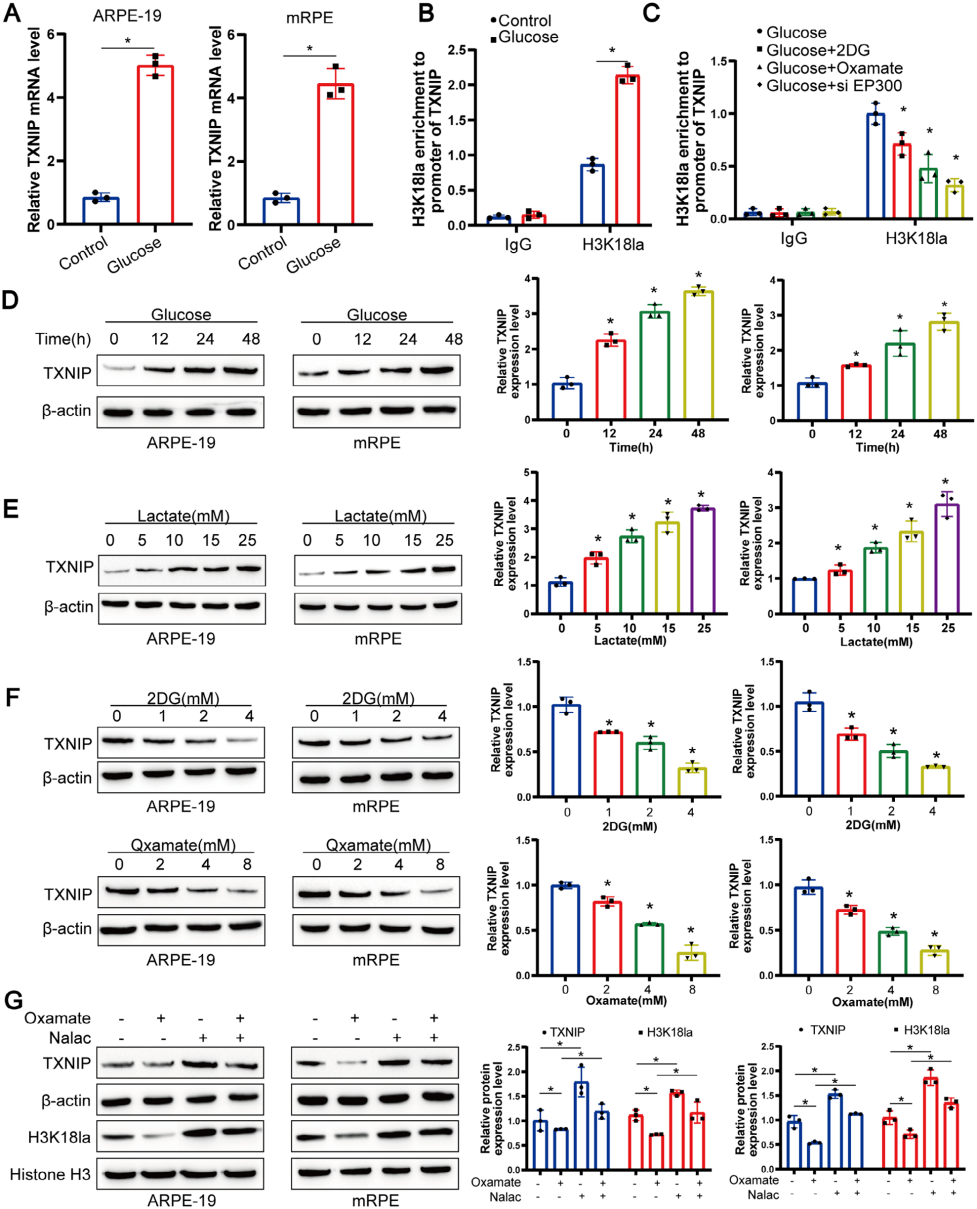
Histone lactylation activates TXNIP transcription in RPE cells. (A) RT‐qPCR analysis of TXNIP mRNA expression in ARPE‐19 and mRPE cells after 48 h of high glucose treatment. β‐Actin was used as internal reference gene. Data represent mean ± SD from three biological replicates (*n* = 3). Statistical significance was determined by one‐way ANOVA followed by Bonferroni post hoc test. *p < 0.05 versus untreated control. (B) ChIP‐qPCR analysis of DNA fragments immunoprecipitated from ARPE‐19 cells using an H3K18la‐specific antibody, followed by qPCR with primers targeting the TXNIP promoter region. Data represent mean ± SD from three biological replicates (*n* = 3). One‐way ANOVA with Bonferroni correction was applied. *p < 0.05 versus control. (C) ChIP‐qPCR assessment of H3K18la enrichment at the TXNIP promoter in ARPE‐19 cells treated with glycolysis inhibitors (4 mM 2‐deoxyglucose [2‐DG], 8 mM oxamate), or transfected with siRNA against EP300. Data represent mean ± SD from three biological replicates (*n* = 3). Statistical analysis was conducted using one‐way ANOVA with Bonferroni post hoc test. *p < 0.05 versus control. (D and E) Representative western blot images showing TXNIP protein expression in ARPE‐19 and mRPE cells treated with high glucose for varying durations (D) and with different concentrations of exogenous lactate (E). β‐Actin served as loading control. Data represent mean ± SD from three biological replicates (*n* = 3). Statistical significance was determined by one‐way ANOVA with Bonferroni correction. *p < 0.05 versus control. (F) Representative western blot images illustrating TXNIP expression levels in ARPE‐19 and mRPE cells treated with increasing concentrations of glycolytic inhibitors (2DG and oxamate). β‐Actin served as loading control. Data represent mean ± SD from three biological replicates (*n* = 3). Statistical analysis was conducted using one‐way ANOVA with Bonferroni post hoc test. *p < 0.05 versus control. (G) Representative Western blot images displaying TXNIP expression in ARPE‐19 and mRPE cells treated with oxamate or supplemented with N‐acetyl‐l‐alanine (Nala). β‐Actin was used as internal control. Data represent mean ± SD from three biological replicates (*n* = 3). Statistical significance was assessed by one‐way ANOVA with Bonferroni correction. *p < 0.05 versus control.

Consistent with expectations, our results revealed a significant upsurge in TXNIP mRNA levels following glucose treatment in RPE cells (Figure 6A). To elucidate the role of histone lactylation in regulating TXNIP gene expression, we employed chromatin immunoprecipitation followed by quantitative PCR (ChIP‐qPCR) using an anti‐H3K18la antibody, focusing on the TXNIP promoter region. This analysis demonstrated a conspicuous peak of H3K18la enrichment at the TXNIP promoter site (Figure 6B). Furthermore, ChIP‐qPCR experiments confirmed that the accumulation of H3K18la at the TXNIP promoter could be attenuated by glycolysis inhibitors or upon knockdown of the histone lactylation enzyme EP300 via siRNA (Figure 6C).

To further validate the regulatory effect of lactate on TXNIP, we conducted in vitro cellular experiments. Specifically, we observed an elevation in TXNIP protein levels in RPE cells cultured with glucose over different time periods or under varying concentrations of lactate (Figures 6D,E). Conversely, treatment with different concentrations of glycolysis inhibitors (2DG and Oxamate) led to a decrease in TXNIP protein levels (Figure 6F). Notably, the decline in TXNIP protein levels induced by Oxamate, a glycolysis inhibitor, could be partially rescued by supplementation with Nala (Figure 6G). Collectively, these data provide robust evidence in favor of a positive regulatory role of H3K18la in TXNIP transcription.

### 2.7 EGR Recruited EP300 Complex to Up‐Regulate the Histone Lactation Level in TXNIP Promoter Region

**FIGURE 7:**
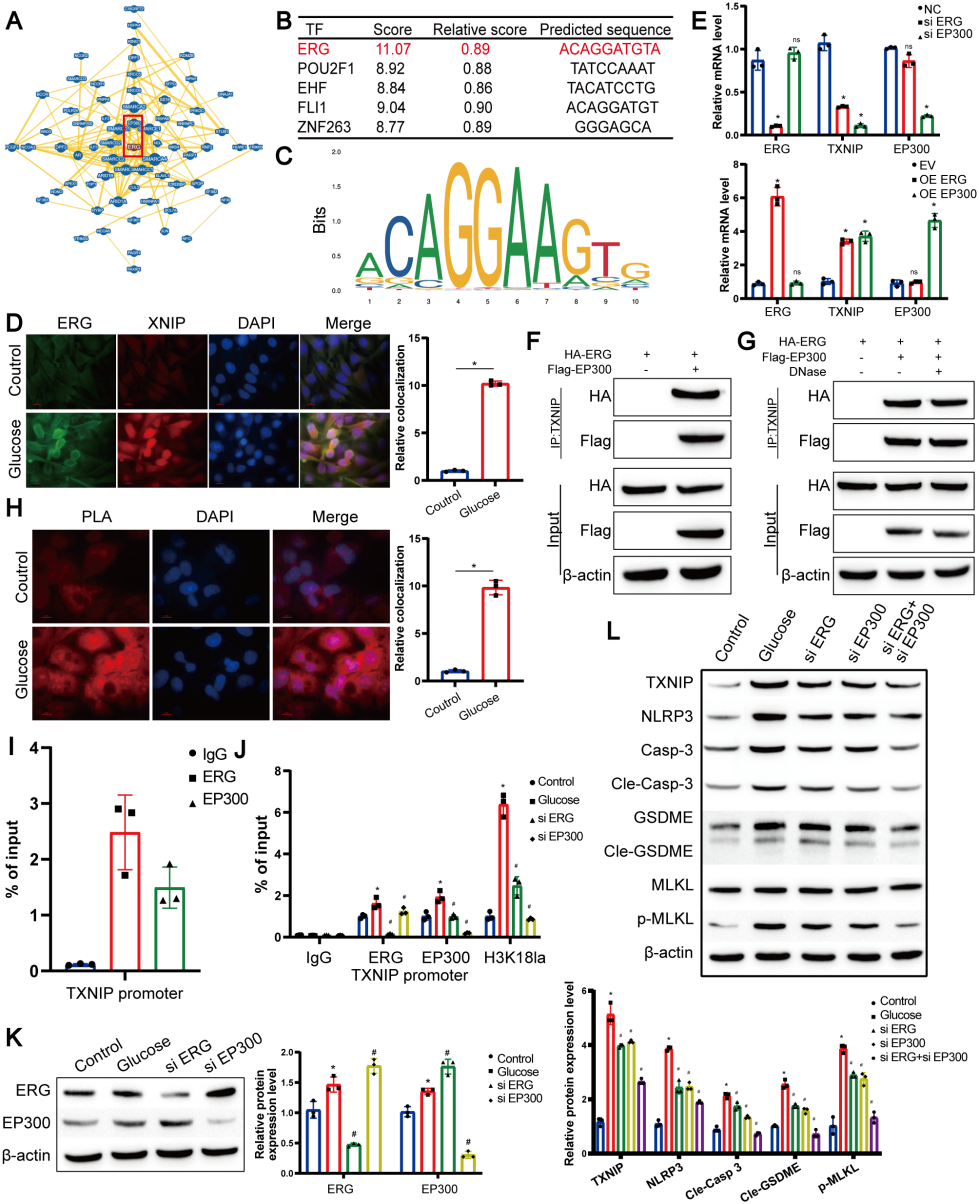
ERG recruits EP300 to form a complex upregulating histone lactylation at the TXNIP Promoter region. (A) Protein–protein interactions between ERG, EP300, and other co‐factors predicted using the BioGRID database. (B and C) Prediction of transcription factors binding to the TXNIP promoter region using the JASPAR database. Putative ERG binding sites are indicated. (D) Representative images of fluorescence in situ hybridization (FISH) showing subcellular localization of TXNIP mRNA in ARPE‐19 cells. Scale bar: 10 µm. (E) qPCR analysis of TXNIP mRNA expression changes following lentiviral‐mediated knockdown or overexpression of ERG and EP300 in ARPE‐19 cells. Data represent mean ± SD from three biological replicates (*n* = 3). Statistical analysis was conducted using one‐way ANOVA with Bonferroni post hoc test. *p < 0.05 versus control. (F–G) Pull‐down assays analyzing direct interaction between ERG and EP300 in ARPE‐19 cell lysates. (H) Representative images of proximity ligation assay (PLA) demonstrating endogenous interaction between ERG and EP300 in ARPE‐19 cells. Scale bar: 10 µm. (I) ChIP‐qPCR assessment of ERG and EP300 occupancy at the TXNIP gene promoter in ARPE‐19 cells. Data represent mean ± SD from three biological replicates (*n* = 3). Statistical significance was determined by one‐way ANOVA with Bonferroni correction. *p < 0.05 versus control. (J) ChIP‐qPCR analysis of ERG, EP300, and H3K18la occupancy at the TXNIP promoter in ARPE‐19 cells stably transfected with siERG or siEP300. Data represent mean ± SD from three biological replicates (*n* = 3). Statistical analysis was conducted using one‐way ANOVA with Bonferroni post hoc test. *p < 0.05 versus control. (K) Representative western blot images showing protein expression levels of ERG and EP300 in stably transfected ARPE‐19 cells. (L) Representative western blot images illustrating expression of PANoptosis‐related proteins (caspase‐3, GSDMD, and pMLKL) in stably transfected ARPE‐19 cells. β‐Actin served as loading control. Data represent mean ± SD from three biological replicates (*n* = 3). Statistical significance was assessed by one‐way ANOVA with Bonferroni correction. *p < 0.05 versus control.

In addition to histones, transcription factors (TFs) play a pivotal role in regulating transcription by binding to gene promoter regions. Notably, the TF ERG has been reported to recruit EP300, a transcriptional co‐activator, to influence leukemia transmission [35]. Furthermore, DLL4 transcription during angiogenesis is reliant on EP300, which interacts with ERG [36]. Using the BioGRID database, we predicted an interaction between EP300 and ERG (Figure 7A). Consequently, we explored whether ERG binds to the TXNIP promoter. Utilizing the JASPAR database, we identified ERG binding to the TXNIP promoter region with the highest score (Figure 7B,C). To validate this prediction, we conducted a FISH assay, revealing increased nuclear localization of ERG protein and TXNIP gene after glucose treatment in RPE cells (Figure 7D). QPCR further confirmed that both ERG and EP300 regulate TXNIP transcription (Figure 7E). Pull‐down analysis reinforced the predicted interaction between ERG and EP300 (Figure 7F), and the addition of DNase did not disrupt this interaction (Figure 7G). Additionally, PLA experiments demonstrated nuclear interaction between ERG and EP300 (Figure 7H). Since ERG and EP300 form complexes, we investigated their recruitment to the TXNIP gene promoter. Results indicated their recruitment to the TXNIP promoter binding site (Figure 7I). ChIP experiments confirmed the association of ERG with EP300 at the corresponding TXNIP binding site (Figures 7J,K). Finally, siRNA‐mediated knockdown of ERG or/and EP300 revealed their influence on PANoptotic proteins (Figure 7L), suggesting that ERG recruits EP300 to the TXNIP promoter, thereby mediating RPE cell PANoptosis through histone modulation of TXNIP.

### 2.8 Glycolysis Mediates RPE PANoptosis via TXNIP Regulation

In our in vivo restorative experiments, we utilized male mice aged around 2 months, administering intraperitoneal injections of STZ dissolved in citrate buffer daily for five consecutive days. Post the final STZ injection, non‐fasting blood glucose levels were monitored at 10 a.m. between days 7 and 15 to ascertain the successful establishment of the diabetic model. Thereafter, mice were therapeutically intervened with intraperitoneal injections of the glycolysis inhibitor 2DG and/or received tail vein infusions of adeno‐associated virus (AAV) carrying *Txnip*‐targeting RNA interference (RNAi).

**FIGURE 8:**
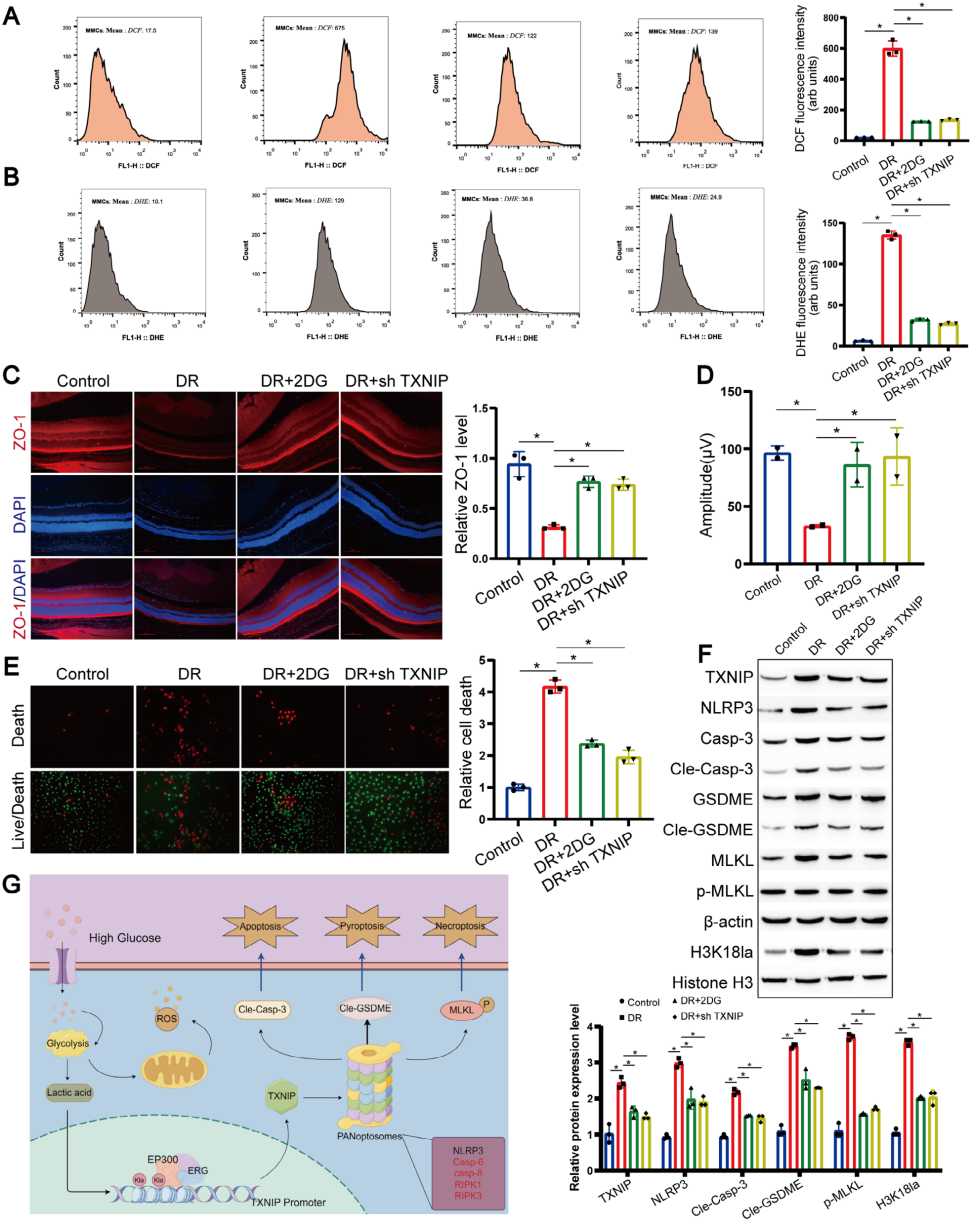
Glycolysis regulates RPE PANoptosis via TXNIP. (A) Quantification of DCF fluorescence intensity indicating ROS levels in ARPE‐19 cells treated with glycolysis inhibitor 2DG and/or TXNIP‐targeting RNAi delivered via adeno‐associated virus (AAV). Data represent mean ± SD from three biological replicates (*n* = 3). Statistical analysis was conducted using one‐way ANOVA with Bonferroni correction. *p < 0.05 versus control. (B) Quantification of DHE staining reflecting superoxide levels in RPE cells under the same treatments. Data represent mean ± SD from three biological replicates (*n* = 3). Statistical significance was determined by one‐way ANOVA with Bonferroni correction. *p < 0.05 versus control. (C) Representative images and quantification of immunofluorescence (IF) staining for cleaved caspase‐3, GSDMD‐N, and pMLKL in retinal sections from mice treated with 2DG and/or AAV‐TXNIP‐RNAi. Scale bar: 100 µm. Data represent mean ± SD from three biological replicates (*n* = 3). Statistical analysis was conducted using one‐way ANOVA with Bonferroni post hoc test. *p < 0.05 versus control. (D) Quantification of ERG a‐ and b‐wave amplitudes under scotopic conditions in mice treated with 2DG and/or AAV‐TXNIP‐RNAi. Data represent mean ± SD from three biological replicates (*n* = 3). Statistical significance was assessed by one‐way ANOVA with Bonferroni correction. *p < 0.05 versus control. (E) Live/Dead viability/cytotoxicity assay using calcein AM (live cells, green) and EthD‐III (dead cells, red) in ARPE‐19 cells treated as above. Scale bar: 100 µm. (F) Representative western blot images showing alterations in PANoptosis‐related protein expressions (caspase‐3, GSDMD, and pMLKL) in RPE cells following treatment with 2DG and/or AAV‐mediated TXNIP knockdown. β‐Actin was used as internal control. Data represent mean ± SD from three biological replicates (*n* = 3). Statistical significance was assessed by one‐way ANOVA with Bonferroni correction. *p < 0.05 versus control. (G) Schematic illustration of the proposed mechanistic pathway linking glycolysis, histone lactylation, ERG‐EP300 complex formation, TXNIP upregulation, and subsequent activation of PANoptosis in RPE cells during diabetic retinopathy.

Our findings illuminated that the application of 2DG alone or in conjunction with *Txnip* knockdown significantly mitigated superoxide levels (Figure 8A) and ROS content (Figure 8B) in the retinal tissues of mice. Through immunofluorescence‐based localization to the RPE, we noted a marked reduction in RPE cell mortality upon treatment with 2DG and/or *Txnip* knockdown (Figure 8C). Further, ERG assessments endorsed enhancements in retinal function (Figure 8D; Figure S1B), implying that 2DG treatment and/or reducing *Txnip* expression facilitate the recuperation of RPE cell performance. Using the LIVE/DEAD Assay Kit, we also observed that treatment with 2DG or/and *Txnip* knockout significantly reduced RPE cell death (Figure 8E). Complementarily, western blot analyses unveiled a profound decrease in the PANoptosis level of RPE cells subjected to 2DG treatment or *Txnip* depletion (Figure 8F).

Collectively, these outcomes accentuate the central role of RPE in DR progression. They imply that RPE cellular impairment stems from chronic hyperglycemia‐induced glycolytic dysregulation, which fosters high lactic acid production, triggers histone lactylation of *Txnip*, activates the *Txnip/Nlrp3* signaling cascade, and eventually instigates PANoptosis (Figure 8G). The efficacy of 2DG and/or *Txnip* modulation in our in vivo models in alleviating this pathological sequence not only confirms the mechanistic link but also charts a promising course for delving deeper into the disease mechanisms of DR and devising innovative therapeutic interventions.

## 3 Discussion

DR is a severe microvascular complication of diabetes mellitus (DM) and the leading cause of blindness among the working‐age population [37, 38]. Hyperglycemia serves as the primary culprit behind DR [39], posing a substantial threat to human health and quality of life. Despite its significance, the pathogenesis of DR remains incompletely understood. Therefore, elucidating the mechanisms underlying DR and discovering novel therapeutic targets are of utmost importance in enhancing the quality of life for patients with DR. Our study underscores the crucial role of the RPE in DR pathogenesis. Specifically, RPE cell death may ensue from aberrant glycolysis, leading to the accumulation of lactic acid. This lactic acid elevation boosts TXNIP milk protein levels within cells, activating the TXNIP/NLRP3 signaling pathway and ultimately triggering PANoptosis. Our in vivo findings reveal that the utilization of 2DG and/or TXNIP knockout can mitigate this process, offering fresh insights into the mechanisms and potential treatments for DR.

RPE cells serve as a crucial component of the blood‐retinal barrier and play a pivotal role in the pathogenesis of DR. In diabetic patients, RPE cells, exposed to a hyperglycemic environment, secrete various factors and signaling molecules that impact ganglion cells, photoreceptor cells, pericytes, and vascular endothelial cells, thereby accelerating the progression of DR [40]. The abnormal metabolism of retinal cells resulting from this hyperglycemic milieu frequently leads to mitochondrial dysfunction, endoplasmic reticulum stress, and other disruptions of cellular homeostasis, ultimately culminating in retinal cell death [41]. Numerous studies have demonstrated a significant increase in RPE cell mortality among DR patients, manifesting in various forms of cell death, including apoptosis, pyroptosis, ferroptosis, and autophagy [42, 43]. However, the precise mechanism underlying these death processes remains incompletely understood. Our findings, employing Western Blot and flow cytometry, reveal that a hyperglycemic environment induces PANoptosis in RPE cells, a process that plays a critical role in DR progression. This PANoptosis, a form of programmed cell death, involves the activation of multiple death pathways simultaneously. Therefore, a thorough investigation of the mechanisms underlying PANoptosis induced by hyperglycemia in RPE cells could provide a valuable theoretical foundation for the discovery of novel diagnostic methods and therapeutic targets for DR. This research avenue holds significant promise for enhancing our understanding and treatment of this debilitating condition.

PANoptosis, a newly emerging concept, sheds light on the intricate crosstalk and coordination among three cellular death pathways: pyroptosis, apoptosis, and necrotic apoptosis [44]. Once PANoptosis is triggered, the blockade of any individual cell death modality (whether pyroptosis, apoptosis, or necrotic apoptosis) is insufficient to halt the inevitable demise of the cell. Given its significance in RPE cells, further elucidating the detailed mechanisms underlying PANoptosis activation holds immense promise for the advancement of future treatments for DR. Prior research has identified NLRP3 as a crucial component of a multiprotein complex that drives PANoptosis [31]. Furthermore, studies have demonstrated that the activation of the mitochondrial ROS‐TXNIP/NLRP3 axis underlies renal tubule injury observed in patients with diabetic nephropathy [32]. This study aims to delve into the regulatory relationship between the TXNIP/NLRP3 signaling pathway and RPE PANoptosis. Utilizing in vitro cell models, we conducted western blot and kit tests, revealing that the activation of TXNIP/NLRP3 signals promotes cellular PANoptosis. This finding offers valuable insights into the pathogenesis of DR and highlights potential therapeutic targets aimed at mitigating RPE cell death and stalling the progression of this debilitating disease.

In the current study, we observed activation of multiple apoptosis‐related caspases, including caspase‐8, ‐9, ‐3, and ‐7, which provides molecular evidence supporting a role for PANoptosis in DR. Caspase‐8 emerged as a key initiator caspase that not only triggers canonical apoptosis but also bridges necroptotic signaling through its interaction with RIPK1 and FADD. Its activation suggests the potential formation of the PANoptosome complex—a multimolecular platform integrating components from apoptosis, pyroptosis, and necroptosis. Meanwhile, the upregulation of caspase‐9 further supports the involvement of the intrinsic mitochondrial apoptotic pathway, reinforcing the notion of a mixed cell death mode rather than isolated apoptosis. Moreover, the robust activation of executioner caspases‐3 and ‐7—commonly associated with terminal apoptotic processes—was also consistent with their recently identified roles in PANoptosis, where they may contribute to the coordinated dismantling of the cell within a broader inflammatory context. Importantly, the simultaneous activation of these caspases across different pathways implies a coordinated engagement of overlapping death mechanisms, rather than independent or sequential activation. This pattern supports the hypothesis that PANoptosis, rather than classical apoptosis alone, contributes to retinal cell death under diabetic conditions. These findings expand our understanding of cell death modalities in DR and suggest that targeting PANoptosis may represent a novel therapeutic strategy for this disease. Another noteworthy aspect is the disruption of mitochondrial balance by long‐term hyperglycemia, leading to the generation of a significant amount of ROS. This, in turn, causes endothelial damage and promotes local hypoxia [33]. Under hypoxic conditions, the majority of eukaryotic cells experience abnormal intracellular glycolysis in a sustained hypoxic environment [34]. Consistent with this, our western blot and kit tests demonstrated abnormal glycolysis in RPE cells and the production of elevated levels of lactic acid in a chronic high‐glucose setting. In recent years, lactate‐triggered histone lysine lactylation has emerged as a novel histone modification. This modification has been implicated in regulating the expression of genes associated with tumorigenesis, progression, and proliferation of cancer cells. Furthermore, it plays a crucial role in maintaining homeostasis gene expression during the polarization of M1 macrophages induced by infection or hypoxia [45]. Here, we present novel findings that histone lactylation regulates RPE PANoptosis by upregulating TXNIP gene transcription and subsequently activating the TXNIP/NLRP3 pathway. This discovery offers new insights into the complex molecular mechanisms underlying RPE cell death and holds promise for the development of targeted therapeutic strategies against DR.

While our data support a role of histone lactylation (H3K18la) in promoting TXNIP transcription, we acknowledge that lactate may also regulate gene expression via non‐epigenetic mechanisms, such as acting as a signaling molecule or through monocarboxylate transporter (MCT)‐mediated entry into cells. Indeed, lactate has been shown to modulate inflammation and oxidative stress through GPR81 signaling and other pathways. Future studies using MCT inhibitors (e.g., AZD3965, an MCT1 inhibitor) or genetic knockdown of lactate transporters could help distinguish between direct epigenetic effects and indirect signaling pathways. One limitation of our current study is the lack of pharmacological or genetic tools to specifically block lactate transport or signaling, which would further clarify the relative contribution of histone lactylation versus metabolite signaling in TXNIP regulation. Our findings suggest that targeting glycolysis or the TXNIP‐NLRP3 axis may offer novel therapeutic avenues for DR. However, systemic administration of glycolytic inhibitors such as 2‐deoxy‐d‐glucose (2DG) carries risks, including impaired energy metabolism in normal tissues and potential neurotoxicity due to its broad cellular uptake. Therefore, localized or targeted delivery methods will be crucial for minimizing off‐target effects. To this end, we propose that tissue‐specific delivery systems, such as AAV vectors with retinal‐specific promoters (e.g., Rho or IRBP), could be used to selectively silence TXNIP in retinal cells. Additionally, nanoparticle‐based or intravitreal delivery of small interfering RNAs or CRISPR tools may offer safer alternatives for modulating this pathway locally.

TFs, apart from histones, bind to gene promoter regions to regulate transcription. These factors often recruit cofactors, such as co‐activators and co‐repressors, to activate or repress target gene transcription [46]. Notably, the TF ERG has been reported to recruit EP300, affecting leukemia transmission [35]. Furthermore, the transcription of DLL4 during angiogenesis relies on the transcriptional coactivator EP300 of ERG. Our experimental findings demonstrated that the EP300 complex recruited by EGR upregulated the histone lactylation level in the TXNIP promoter region, thus promoting TXNIP transcription. This discovery offers a robust molecular mechanism explanation for the PANoptosis of RPE in DR.

While this study delineates a novel mechanistic link between glycolytic‐driven histone lactylation, TXNIP/NLRP3‐mediated PANoptosome formation, and PANoptosis in DR pathogenesis using robust in vitro and in vivo models, a significant limitation is the lack of validation in clinical human samples. Our findings in human RPE cells under high glucose and diabetic mouse models provide compelling preclinical evidence; however, direct confirmation in retinal tissues or vitreous samples from DR patients is absent. Future studies should assess the relevance of this glycolysis‐histone lactylation‐TXNIP/NLRP3‐PANoptosis axis in clinical specimens to verify its translational significance and potential as a therapeutic target in human disease progression. This clinical correlation is essential to bridge our mechanistic insights to real‐world patient applications. Another limitation of the current study is that our mechanistic investigations were primarily conducted in RPE cells. While these cell types are central to the pathogenesis of DR, the involvement of other relevant cell types—such as retinal endothelial cells, astrocytes, and microglia—remains to be determined. Given that glycolytic reprogramming and inflammasome activation are common features across multiple retinal cell types under diabetic conditions, it will be important to examine whether the TXNIP‐NLRP3‐mediated PANoptotic mechanism operates similarly in these cells.

## 4 Conclusion

Our investigation reveals a pivotal role for dysregulated glycolysis in the pathogenesis of DR, specifically through histone lactylation‐mediated TXNIP activation in RPE cells. This metabolic disturbance induces PANoptosis, a multi‐modal cell death process, within RPE, exacerbating retinal damage. The study underscores the therapeutic potential of targeting glycolysis and TXNIP as interventions mitigated RPE PANoptosis. Our findings suggest that modulating these pathways holds promise for preserving RPE integrity and halting DR progression, highlighting a critical metabolic‐epigenetic axis in disease management. Further research is warranted to translate these insights into effective clinical therapies.

## 5 Methods

### 5.1 Animal

C57BL/6J mice were obtained from Slaccas (Shanghai, China) and were handled in accordance with the ARVO's Statement for the Use of Animals in Ophthalmic and Vision Research, as well as the Animal Care and Use Guidelines issued by the National Research Council. All experimental protocols were approved by the Institutional Animal Care and Use Committee of the First Affiliated Hospital of Kunming Medical University (IACUC No. kmmu20230619).

### 5.2 Induction of Diabetes

Male mice (8‐week‐old) were intraperitoneally injected with streptozotocin (STZ, Sigma‐Aldrich, #S0130; 60 mg/kg/day × 5 days) dissolved in cold citrate buffer (pH 4.5, injection volume ≤ 200 µL). Non‐fasting blood glucose was monitored daily from day 7 to 15 post‐injection using a glucometer (Accu‐Chek). Diabetes was confirmed when mice exhibited sustained hyperglycemia (> 275 mg/dL) on three consecutive measurements. HbA1c levels were quantified via ELISA (Crystal Chem #80310) at sacrifice. Following diabetes confirmation, diabetic mice were randomly assigned to different treatment groups. For glycolysis inhibition, mice were intraperitoneally administered 2DG (Sigma‐Aldrich, D6134) at a dose of 200 mg/kg body weight, once daily for 14 consecutive days. For TXNIP knockdown experiments, a lentivirus‐based shRNA targeting TXNIP (shTXNIP, sequence: GCAAGTAGCATCTCTGATT) was delivered via intravitreal injection (1 µL per eye, titer ∼1 × 10⁹ TU/mL). Animals were analyzed 7 days post‐injection to assess the effects of TXNIP silencing on retinal pathology and inflammation. To prevent severe catabolism during STZ‐induced diabetes, low‐dose insulin glargine (0–0.2 IU/day, Thermo #12585014) was subcutaneously administered based on weekly glycemic and weight monitoring (two to three times weekly).

### 5.3 Bioinformatics Prediction

The Biological Universal Repository of Interaction Datasets (BioGRID) is a public database for archiving and disseminating genetic and protein interaction data from model organisms and humans (https://thebiogrid.org/). JASPAR A database that collates TF binding profiles and can be used to scan any DNA sequence to predict TF‐binding sites (TFBS). (https://jaspar.elixir.no/).

### 5.4 Statistical Analysis

Data normality was assessed via Shapiro–Wilk test (*α* = 0.05). Parametric data (mean ± SD) were analyzed by two‐tailed Student's *t*‐test (two groups) or one‐way ANOVA with Tukey correction (≥ 3 groups). Non‐parametric data used Mann–Whitney *U* or Kruskal–Wallis/Dunn's tests. Survival curves employed log‐rank tests. All analyses used GraphPad Prism 9.0 with Benjamini–Hochberg FDR correction (*q* < 0.05 considered significant). Power analysis (*β* = 0.2, *α* = 0.05) determined minimum *n* = 5/group.

Other methods are provided in the Supporting Information.

## Author Contributions

Xiaoting Xi, Qianbo Chen, and Jia Ma contributed to the conception. Xuewei Wang and Yuxin Zhang contributed to the design. Qiuxia Xiong, Xiaolei Liu, and Yuan Xia analyzed and interpreted the data. Xiaoting Xi drafted the article. Yan Li critically revised it for important intellectual content. All authors approved the final version for publication.

## Ethics Statement

This study and included experimental procedures were approved by The First Affiliated Hospital of Kunming Medical University. All experimental protocols were approved by the Institutional Animal Care and Use Committee of the First Affiliated Hospital of Kunming Medical University (IACUC No. kmmu20230619).

## Conflicts of Interest

The authors declare no conflicts of interest.

## Consent

The authors have nothing to report.

## Supporting information



Supporting Information

## Data Availability

The datasets used and/or analyzed during the current study are available from the corresponding author on reasonable request.
