# Supplementary material for: Glycolysis‐Histone Lactylation Crosstalk Drives TXNIP‐NLRP3‐Mediated PANoptosome Assembly and PANoptosis Activation Underlying Diabetic Retinopathy Pathogenesis
Source: MedComm (2020). 2025 Sep 14;6(9):e70351. doi: 10.1002/mco2.70351 (PMC12433895; doi:10.1002/mco2.70351)
Supplement: Supplementary file 1 — Supporting Information [file MCO2-6-e70351-s001.docx]

**Title page**

**Title:** **Glycolysis-Histone Lactylation Crosstalk Drives TXNIP-NLRP3-Mediated PANoptosome Assembly and PANoptosis Activation Underlying Diabetic Retinopathy Pathogenesis**

**Running title:** Glycolysis induces PANoptosis in RPEc

Xiaoting Xi^1^, Qianbo Chen^1^, Jia Ma^1^, Xuewei Wang^1^, Yuxin Zhang^1^, Qiuxia Xiong^2^, Xiaolei Liu^3^, Yuan Xia^1^, Yan Li ^1*^

^1^ Ophthalmology Department, The First Affiliated Hospital of Kunming Medical University, Kunming City, 650032, China.

^2^ Yunnan Key Laboratory of Laboratory Medicine, The First Affiliated Hospital of Kunming Medical University, Kunming City, 650032, China.

^3^ Neurology Department, The First Affiliated Hospital of Kunming Medical University, Kunming City, 650032, China.

**^*^Corresponding author:** **Yan Li**, Ophthalmology Department, The First Affiliated Hospital of Kunming Medical University, 295 Xichang Road, Kunming City, Yunnan Province, 650032, China.

**Email:** xi_xiao_ting@163.com

**
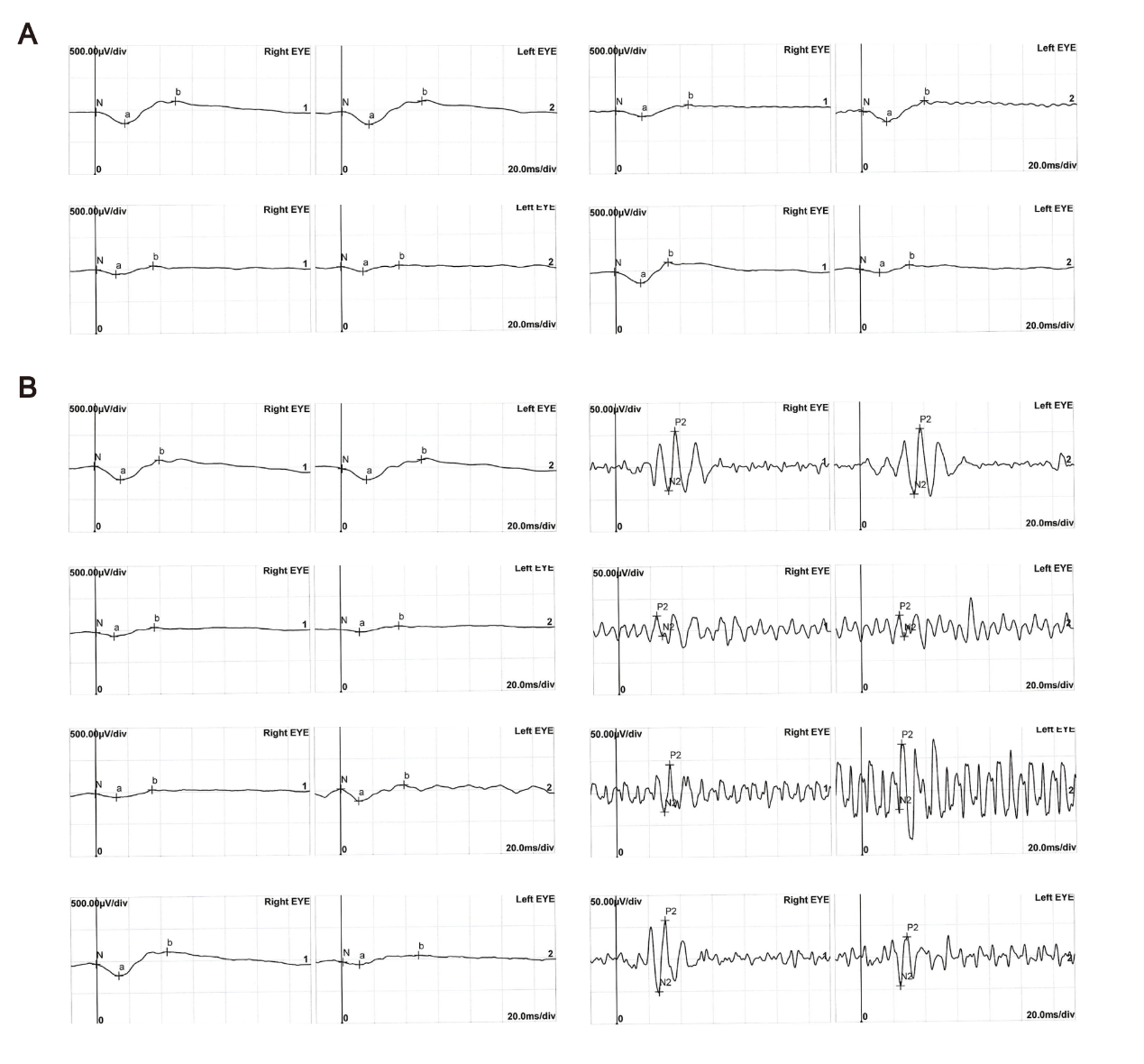
**

**Figure S1 Electroretinogram of DR Mouse**

(A) Scotopic and photopic electroretinograms (ERG) recorded from control mice and diabetic mice induced by streptozotocin (STZ). Representative waveforms and quantification of a-wave and b-wave amplitudes are shown.

(B) ERG responses were assessed in the DR model group following intravitreal injection of 2-deoxyglucose (2DG) or shTXNIP-expressing lentivirus. Quantitative analysis of a-wave and b-wave amplitudes demonstrates functional improvement in retinal responses.

**
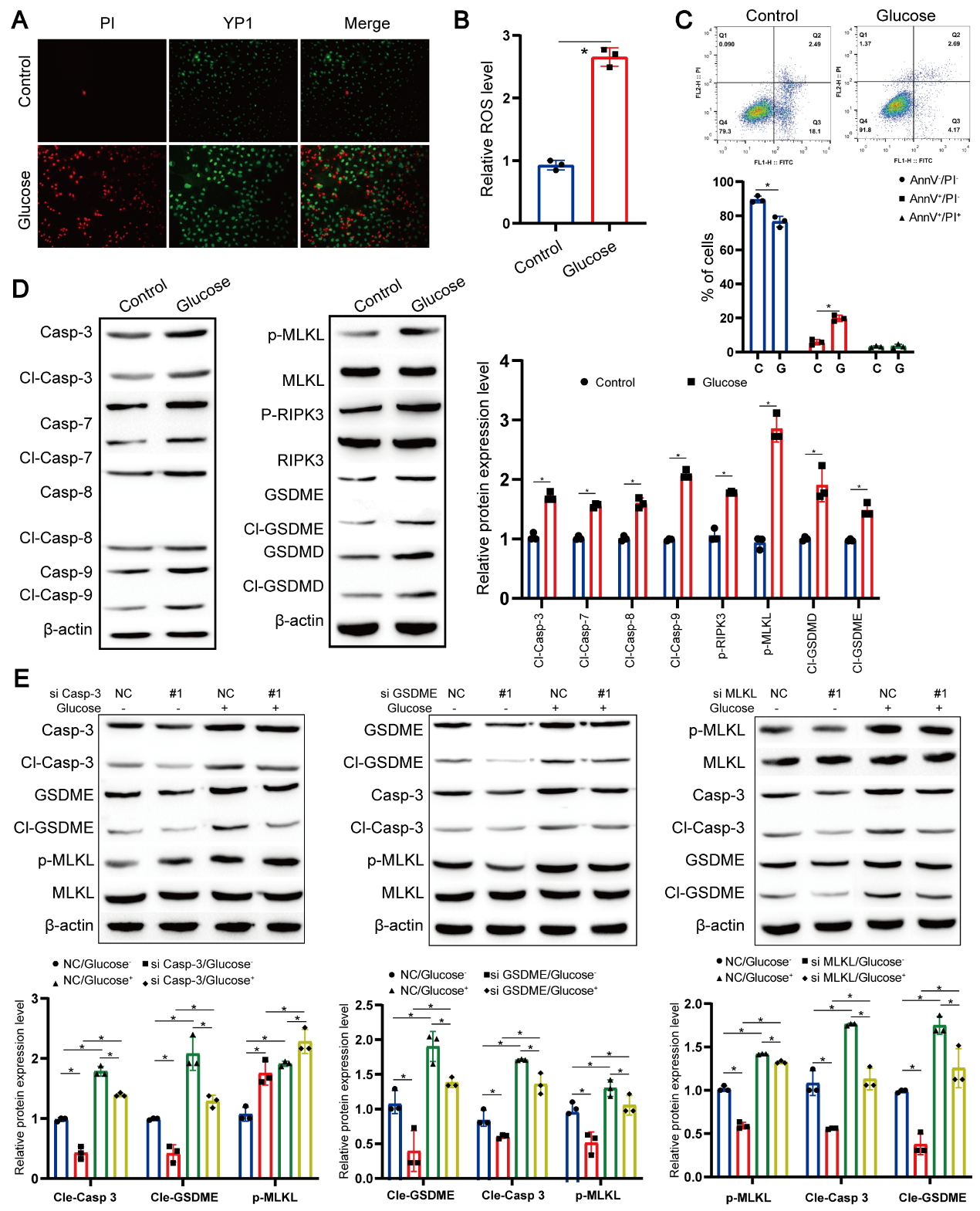
**

**Figure S2 Characterization of PANoptosis in RPE Cells under High Glucose Conditions**

(A) Representative fluorescence images showing YP1-positive (green, pyroptotic marker) and propidium iodide (PI)-positive (red, necrotic cell death) staining in ARPE-19 cells after high glucose treatment. Scale bar = 100 μm.

(B) Quantification of reactive oxygen species (ROS) levels using DCFDA staining. Data represent mean ± SD from three independent experiments (n = 3). One-way ANOVA with Bonferroni correction was used. *p < 0.05 vs. control.

(C) Flow cytometry analysis of Annexin V/PI-stained cells to assess apoptosis and necrosis in RPE cells treated with high glucose for 48 hours. Quantitative data show increased percentages of early and late apoptotic/necrotic cells.

(D) Representative Western blot images showing expression levels of key proteins involved in apoptosis (caspase-3, -7, -8, -9 and their cleaved forms), necroptosis (phosphorylated MLKL, total MLKL, phosphorylated RIPK3, total RIPK3), and pyroptosis (GSDME, GSDMD and their cleaved forms) in RPE cells after 48 hours of high glucose exposure. β-actin served as loading control.

(E) Western blot analysis of phosphorylated MLKL, total MLKL, caspase-3, cleaved caspase-3, GSDME, and cleaved GSDME in ARPE-19 cells following siRNA-mediated knockdown of caspase-3, GSDME, or MLKL.

Data represent mean ± SD from three biological replicates (n = 3). Statistical significance was calculated using one-way ANOVA with Bonferroni correction. *p < 0.05 vs. control.

**
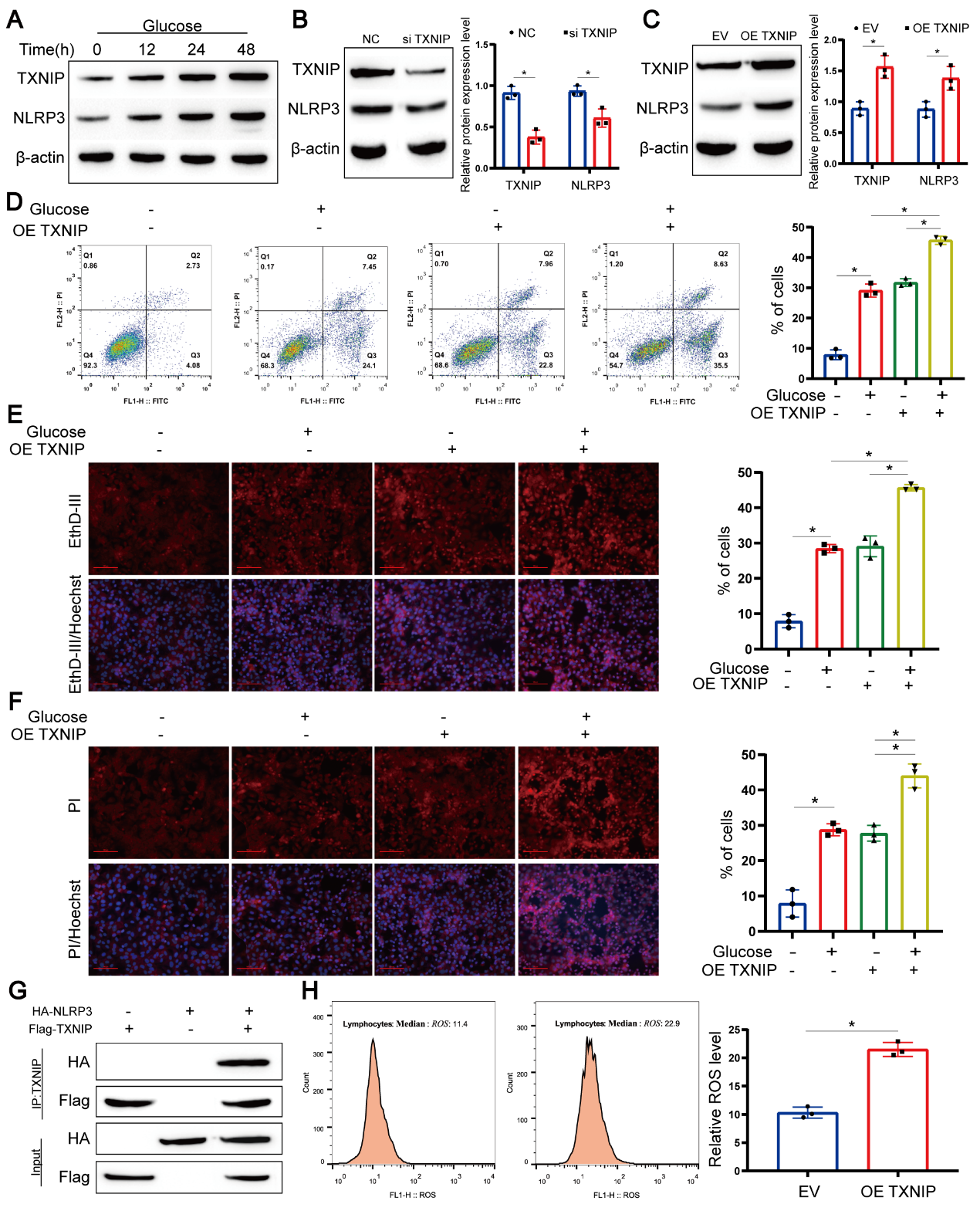
**

**Figure S3 TXNIP/NLRP3 Pathway Regulates PANoptosis in RPE Cells**

(A) Representative Western blot images showing activation of TXNIP/NLRP3 signaling pathway in murine RPE (mRPE) cells after high glucose treatment.

(B) Representative Western blot images demonstrating effective knockdown of TXNIP by specific siRNAs.

(C) Representative Western blot images illustrating overexpression of TXNIP (OE TXNIP) in ARPE-19 cells.

(D–F) Representative immunofluorescence images depicting apoptosis (TUNEL staining, D), pyroptosis (GSDMD-N staining, E), and necroptosis (pMLKL staining, F) in RPE cells treated with high glucose alone or in combination with TXNIP overexpression. Scale bar = 100 μm.

(G) Pull-down assay confirming direct interaction between TXNIP and NLRP3 in RPE cells.

(H) Quantification of ROS levels using DCFDA staining in RPE cells treated with high glucose and/or TXNIP overexpression. Data represent mean ± SD from three biological replicates (n = 3). Statistical analysis was performed using one-way ANOVA with Bonferroni post hoc test. *p < 0.05 vs. control.

**
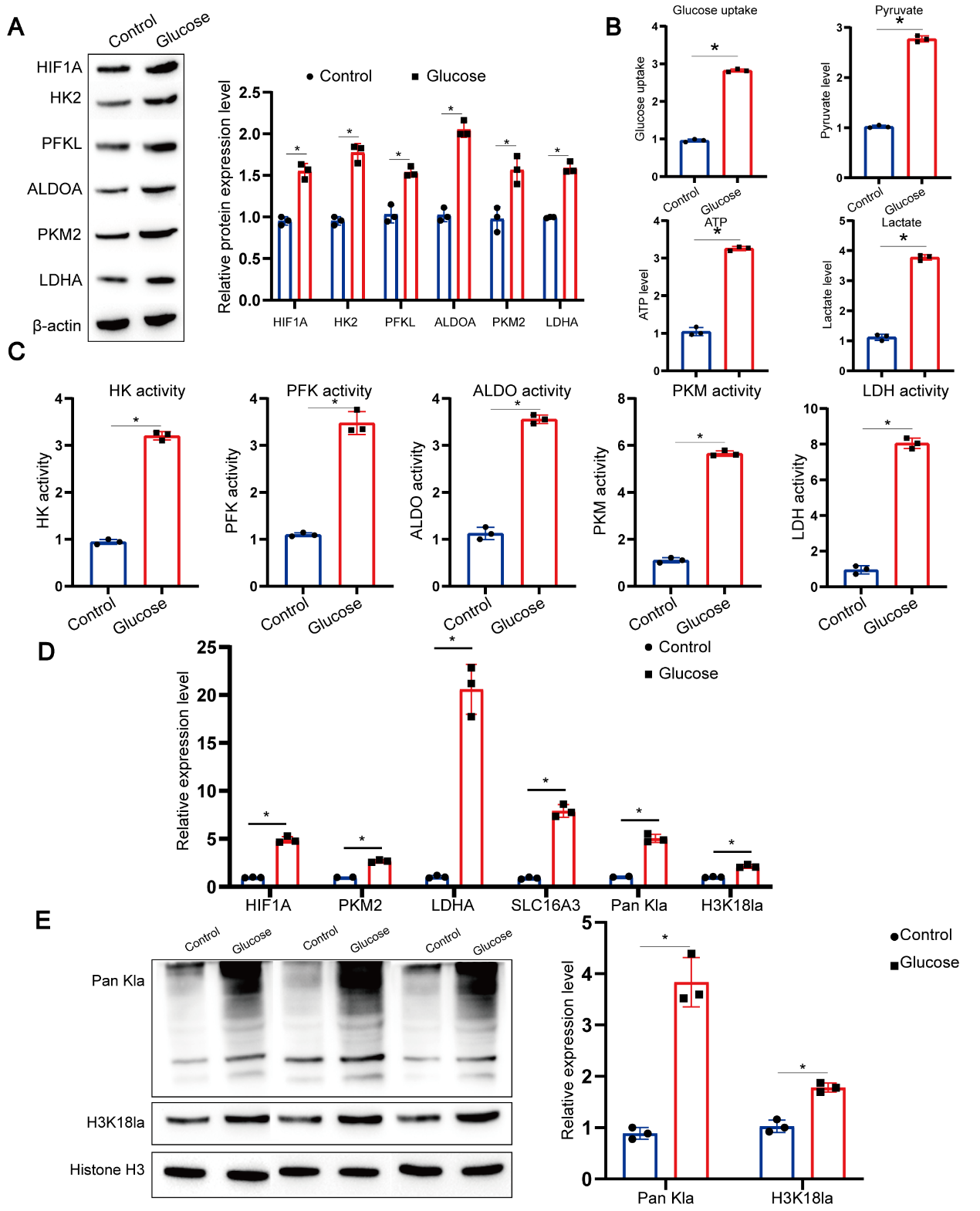
**

**Figure S4 Glycolytic Dysregulation Induces Histone Lactylation in RPE Cells**

(A) Representative Western blot images showing changes in HIF1A and glycolytic enzymes (HK2, PFKL, ALDOA, PKM2, LDHA) in ARPE-19 cells after high glucose treatment. β-actin served as loading control.

(B–C) Quantitative measurements of glucose uptake, pyruvate production, lactate accumulation, ATP content, and enzymatic activities of hexokinase (HK), phosphofructokinase (PFK), aldolase (ALDO), pyruvate kinase (PKM), and lactate dehydrogenase (LDH) in RPE cells. Data represent mean ± SD from three biological replicates (n = 3). One-way ANOVA with Bonferroni correction was applied. *p < 0.05 vs. control.

(D) Quantification of immunohistochemistry (IHC) signals for HIF1A, PKM2, LDHA, SLC16A3 (MCT4), and histone lactylation (H3K18la) in retinal tissues from diabetic and control mice. Data represent mean ± SD (n = 3). One-way ANOVA with Bonferroni post hoc test was used. *p < 0.05 vs. control.

(E) Representative Western blot images assessing global histone lactylation levels in RPE cells after high glucose exposure. Histones were extracted and probed with anti-H3K18la antibody. Data represent mean ± SD from three biological replicates (n = 3). Statistical significance was determined by one-way ANOVA with Bonferroni correction. *p < 0.05 vs. control.

**
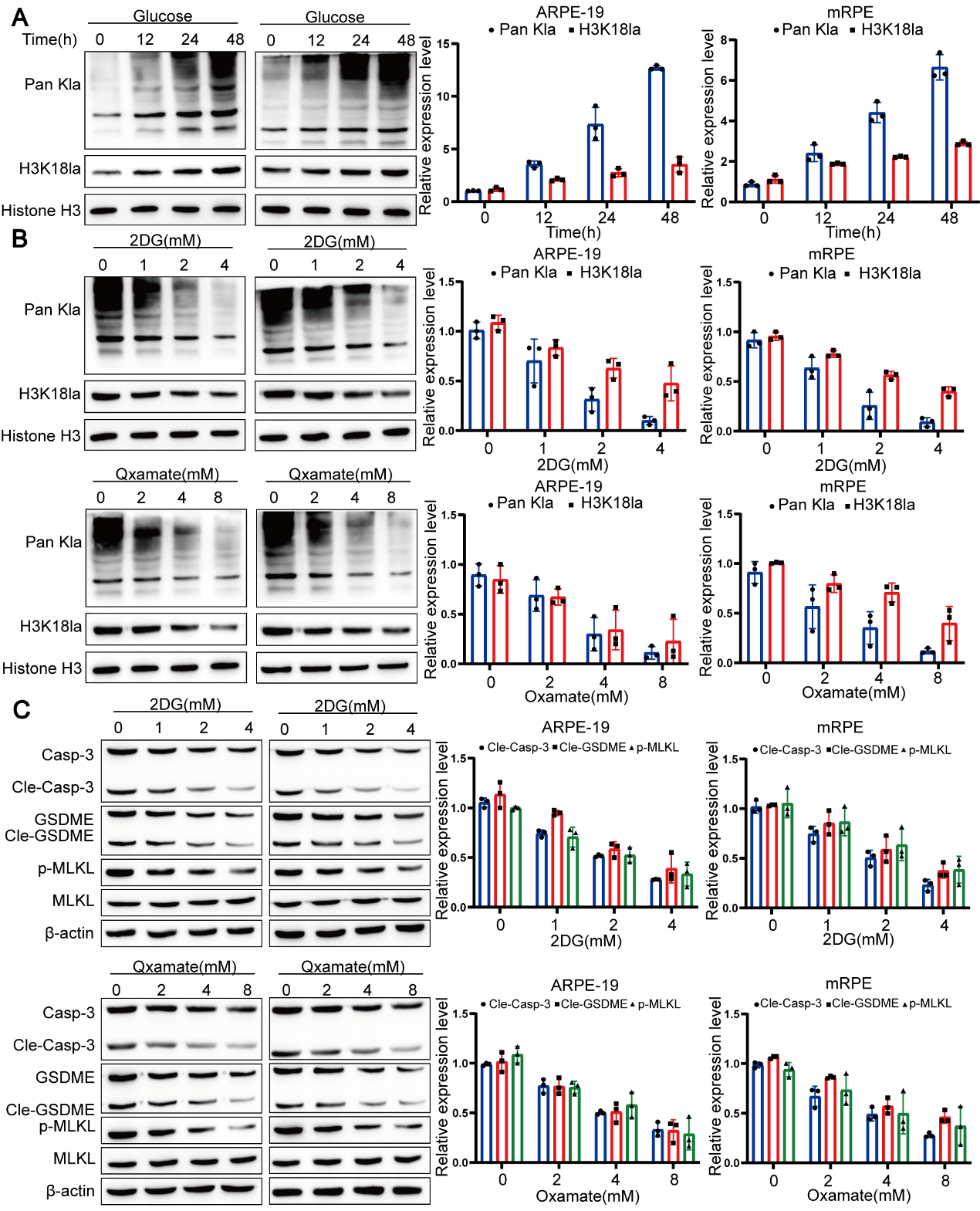
**

**Figure S5 Abnormal Glycolysis Induces PANoptosis of RPE Cells**

(A) Representative Western blot images showing time-dependent changes in histone lactylation (H3K18la) levels in RPE cells exposed to high glucose for varying durations (0, 12, 24, 48 hours). Histone extraction was performed and probed with H3K18la-specific antibody.

(B) Representative Western blot images showing reduced histone lactylation levels in RPE cells treated with increasing concentrations of glycolytic inhibitors (2DG and oxamate).

(C) Representative Western blot images illustrating altered expression of PANoptosis-related proteins (caspase-3, cleaved caspase-3, GSDMD-N, pMLKL) in RPE cells following glycolytic inhibition. β-actin served as internal control.

Data represent mean ± SD from three biological replicates (n = 3). Statistical analysis was conducted using one-way ANOVA with Bonferroni post hoc test. *p < 0.05 vs. control.

**Antibodies and reagents**

The antibodies used for western blotting were:Phospho-MLKL (ab196436, 1:1000), MLKL (ab184718, 1:1000), RIP1 (ab125072, 1:1000), GSDME (ab215191, 1:1000) from Abcam (Cambridge, MA, USA);Caspase-3 (9662S, 1:1000), Cleaved-Caspase-3 (96664S, 1:1000), Caspase-7 (12828S, 1:1000),Cleaved-Caspase-7 (8438S, 1:1000), Caspase-8 (8592S,1:1000), Caspase-9 (9502P, 1:1000), Cleaved-Caspase-9 (7237S, 1:1000), GSDMD (93709S,1:1000)from CellSignaling Technology (Beverly, MA, USA); Flag-tag (F1804, 1:2000) from Sigma-Aldrich (St. Louis, MO, USA); Phospho-RIP1 (28252-1-AP, 1:1000) from Proteintech (Wuhan, China). Primary antibodies used in this study are used in this study are anti-Pan Kla (Jingjie, PTM-1401RM), anti-H3K18la (Jingjie, PTM-1406RM),anti-HIF1A (Abcam, ab179483), anti-Histone H3 (Cell Signaling Technology, 4499), anti-LDHA (Proteintech, 19987–1-AP),anti-EP300 (Abcam, ab275378), anti-FLAG tag (Proteintech, 60002–1-Ig), anti-HA tag (Proteintech, 51064–2-AP).

Anti-His (27-4710-01) antibodies were obtained from GE Healthcare (Chicago, IL). Anti -ALDOA (11217-1-AP), anti-LDHA (19987-1AP) and anti-HIF1𝛼 (20960-1-AP) were obtained from Proteintech (Chicago, IL). Anti-PKM2 (4053S) and anti-HK2 (2867S) were purchased from Cell Signaling Technology (Danvers, MA).

Z-VAD(OH)-FMK (161401-82-7), 3-methyladenine (5142-23-4), necrostatin-1 (4311-88-0), and disulfiram (T0054) were obtained from TargetMol (Shanghai, China); Ferrostatin-1 (347174-05-4) was procured from MedChemExpress (NJ, USA). Ac-DMPD/DMLD-CMK was kindly provided by professor Haiping Hao (China Pharmaceutical University, Nanjing, China). 2-Deoxy-D-glucose(Sigma-Aldrich, D6134),Oxamate(HY-W013032A) and Lactate(HY-B2227) was procured from MCE (Shanghai, China)

**Method**

**5.1 Immunofluorescence Analysis**Retinal cryosections and ARPE-19 cells were fixed in 4% paraformaldehyde (4°C, 12 h) followed by permeabilization with 0.5% Triton X-100/PBS (10 min). After blocking with 3% BSA/PBS (1 h), samples were incubated with primary antibodies (4°C, 16 h; negative controls without primary antibody) and species-matched fluorescent secondary antibodies (RT, 1 h). Nuclei were counterstained with DAPI (5 μg/mL, 5 min) before mounting with Dako fluorescent mounting medium. Confocal imaging was performed using a Nikon A1R system with consistent exposure parameters across experimental groups. Z-stack images (0.5 μm intervals) were acquired using a 60× oil-immersion objective.

**5.2 Reactive Oxygen Species Detection**Retinal superoxide production was quantified using lucigenin-enhanced chemiluminescence. Freshly isolated retinas were equilibrated in Krebs-Hepes buffer (5/25 mM glucose, 37°C, 5% CO₂, 5 min) followed by 0.54 mM lucigenin incubation (5 min). Chemiluminescence was measured using a GloMax 20/20 luminometer (integration time: 10 s) and normalized to tissue protein content.
For spatial ROS localization, unfixed 12 μm retinal cryosections (OCT-embedded, dry ice-frozen) were acetone-fixed (-20°C, 10 min) and stained with 10 μM DCF-DA (60 min dark) or 0.625 μM DHE (20 min dark). After PBS washing, slides were mounted with DAPI Fluoromount-G and imaged on a Zeiss LSM 710 confocal microscope. DCF fluorescence (488/525 nm) was quantified in photoreceptor segments, while DHE signal (535/610 nm) was measured in ONL/INL nuclei using Zen software.

**5.3 Electroretinography (ERG)**Retinal function was assessed at 4-month post-diabetes onset (6-month-old mice) using full-field ERG (Diagnosys Celeris System). After overnight dark adaptation (>12 h), mice were anesthetized (ketamine/xylazine: 87.5/12.5 mg/kg i.p.) with corneal analgesia (0.5% proparacaine) and mydriasis (2.5% phenylephrine). Body temperature was maintained at 37°C using a heating platform. Responses were recorded via corneal electrodes (Burian-Allen) with subcutaneous reference (nasal) and ground (tail) electrodes. Stimuli included dark-adapted (0.01-10 cd·s/m²) and light-adapted (30 cd/m² background) flashes. a-wave amplitude (baseline to trough), b-wave amplitude (a-wave trough to peak), and c-wave (2.5 cd·s/m² pre-light adaptation) were analyzed using Espion V6 software with 5-1000 Hz bandpass filtering.

**5.4 Retinal Leukostasis Quantification**Intracardiac perfusion was performed under deep anesthesia using heparinized saline (37°C, 2 min) followed by FITC-conjugated Concanavalin A (ConA, Vector Labs #FL-1001; 1:50 in PBS, 10 mL/min ×1 min). After ocular enucleation, retinas were flat-mounted in Fluoromount-G and imaged within 2 h using a Zeiss LSM 710 confocal system (20× objective, 488 nm excitation). Adherent leukocytes (>10 μm diameter, FITC⁺/DAPI⁺) were quantified across four retinal quadrants using ImageJ particle analysis (threshold: 50-255 intensity).

**5.5 Retinal Acellular Capillary Quantification**Eyes from 8-month diabetic and age-matched control mice were immersion-fixed in 4% paraformaldehyde (Alfa Aesar #J61899-AP, 48 h, RT). Retinas were dissected, digested with 40 U/mL pronase (Millipore #324682, 37°C, 2 h), and cleared in Tris-HCl (pH 8.5, 16 h). Neuronal tissue was mechanically removed using a 20G brush under stereomicroscopic guidance. Isolated vasculatures were flat-mounted, air-dried (24 h), and stained with hematoxylin (Fisher #SH26-500D)/PAS (Fisher #SS32-500). Acellular capillaries (nucleus-free vessel segments >20 μm) were quantified in six central retinal fields (0.5 mm²/field) using a Nikon Eclipse 90i microscope (40× objective, NIS-Elements AR 5.21 software).

**5.6 Live/dead cell assay**

ARPE-19 cells seeded in 24-well plates (5×10⁴ cells/well) were stained using the LIVE/DEAD Viability/Cytotoxicity Kit (Thermo #L3224). Viable cells (calcein-AM⁺, 494/517 nm) and dead cells (ethidium homodimer-1⁺, 528/617 nm) were imaged with a Zeiss Axio Observer 7 fluorescence microscope (10× objective). Five random fields/well were analyzed using ImageJ FIJI (threshold: 50-255 intensity), with viability calculated as [(live cells)/(live+dead cells)] ×100%.

**5.7 Cell Viability and Cytotoxicity Assessment**ARPE-19 cells were seeded in 96-well plates (NEST Biotechnology, 2×10⁴ cells/well) and treated with experimental compounds for 24 h. Cell viability was quantified using the MTS assay (Promega #G3580): 20 μL reagent/well incubated for 2 h at 37°C, with absorbance measured at 490 nm (Biotek Synergy H1, reference wavelength 630 nm). For cytotoxicity evaluation, supernatant was centrifuged (300 ×g, 5 min) to remove debris prior to LDH quantification using the Roche kit (#11644793001). Reaction mixtures (100 μL supernatant + 100 μL LDH reagent) were incubated for 30 min at RT, terminated with 1N HCl, and read at 490 nm.

**5.8 Apoptosis and ROS Analysis by Flow Cytometry**High glucose-exposed RPE cells were harvested using EDTA-free trypsin, washed with cold PBS, and dual-stained with Annexin V-FITC/PI (KeyGEN, 10 μL each reagent/10⁶ cells, 15 min dark). For ROS detection, cells were loaded with 10 μM CM-H2DCFDA (Thermo #C6827) in serum-free medium (37°C, 30 min dark). Flow cytometry was performed on a Beckman Coulter CytoFLEX system (488 nm laser) analyzing 10,000 events/sample. Apoptotic populations were gated as Annexin V⁺/PI⁻ (early apoptosis) and Annexin V⁺/PI⁺ (late apoptosis). ROS levels were quantified as geometric mean fluorescence intensity (FITC channel) using FlowJo v10.8 software.

**5.9 Western blotting**

The protein was extracted using RIPA buffer (P0013B, Beyotime) and then separated by SDS-PAGE. Subsequently, it was transferred to a PVDF membrane (IPVH00010, Bio-Rad Laboratories, Hercules, CA, USA). The membrane underwent blocking at room temperature for 1 hour, followed by incubation with the primary antibody at 4°C overnight. This was followed by incubation with the peroxidase-conjugated secondary antibody (ZB-2301, ZSBG-Bio) at room temperature for 1 hour. Bands were visualized through chemiluminescence experiments using reagents from Thermo Fisher Scientific (34096). This entire protocol enabled the specific detection and quantification of target proteins, providing valuable insights into their expression and potential roles in cellular processes. See **Supplementary** for antibodies.

**5.10 Cell Lines, Transfection, and Infection**

HEK293T and ARPE-19 cells (Wuhan Punosai Life Technology, mycoplasma-free) were maintained in DMEM/F12 (Gibco) with 10% FBS (Gibco) at 37°C/5% CO₂. For transient transfection, cells at 70% confluency were transfected with plasmid DNA (2 μg/well, Lipofectamine 3000) or siRNA (50 nM, Lipofectamine RNAiMAX) following manufacturer protocols. For sequential siRNA/plasmid transfection, cells were first transfected with siRNA, followed by plasmid transfection 24 h later. Lentiviral particles were produced by co-transfecting HEK293T cells with pPACK packaging mix (System Biosciences) and transfer vectors (MOI=10) using Megatran (Origene). Viral supernatants were harvested at 48 h, concentrated via PEG-it (System Biosciences), and titrated by qPCR.

**5.11 Genetic Constructs**TXNIP promoter-driven luciferase reporters were generated by cloning genomic fragments (-1500 to +100 bp) into pGL4.10 (Promega). FLAG/HA-tagged expression vectors were constructed by inserting PCR-amplified ORFs (primers in Table S1) into pcDNA3.1(+) (Invitrogen). shRNA sequences targeting TXNIP (Table S1) were cloned into pSIH-H1-Puro (System Biosciences). All constructs were validated by Sanger sequencing (TSINGKE).

**5.12 Quantitative PCR**Total RNA extracted with TRIzol (Thermo #15596018) was reverse-transcribed using PrimeScript RT Master Mix (Takara #RR036A). qPCR was performed on a LightCycler 480 II (Roche) with GoTaq qPCR Master Mix (Promega #A6002): 95°C/2 min, 40 cycles of 95°C/15 s, 60°C/30 s. β-actin served as endogenous control. Relative expression was calculated by 2^(−ΔΔCt). Primers are shown in Table S2.

**5.13 Pyroptosis Detection**RPE cells were stained with 1 μg/mL EthD-III (Biotium #40050, 10 min, RT) in PBS, fixed with 4% PFA (20 min), and counterstained with 1 μg/mL Hoechst 33258 (Sigma #94403). Six random fields per sample were imaged under a Zeiss Axio Observer Z1 microscope (20× objective). EthD-III⁺ cells (red fluorescence, 550/620 nm) were quantified using ImageJ (threshold: 50-255 intensity) and normalized to total nuclei.

**5.14 Necrotic Apoptosis Detection by Propidium Iodide (PI) Staining**High glucose/siTXNIP-treated RPE cells were washed with PBS (3×5 min) and stained with 5 μg/mL PI (Sigma #P4170) in PBS (25°C, 20 min dark). After fixation with 4% PFA (RT, 20 min), nuclei were counterstained with 0.5 μg/mL Hoechst 33258 (Sigma #94403, 10 min). Cells were mounted in glycerol/PBS (1:9) and imaged using a Leica DMi8 fluorescence microscope (10× objective). PI⁺ cells (red fluorescence, 535/617 nm) in six random fields were quantified via ImageJ (v1.53) using particle analysis (size: 10-100 μm²; circularity: 0.6-1.0).

**5.15 Protein Interaction Analysis***RPE cells (5×10⁶) were crosslinked with***:**Cells were lysed in Tris-NP40 buffer (50 mM Tris pH 8.0, 500 mM NaCl, 0.5% NP-40, 1 mM DTT, protease inhibitors). Lysates (500 μg) were incubated with anti-FLAG M2 agarose (Sigma #A2220) or anti-HA beads (Pierce #26181) at 4°C for 16 h. Beads were washed (3×1 mL lysis buffer) and bound proteins eluted with 2× Laemmli buffer for WB analysis.
**His Pull-Down:**His-tagged proteins expressed in E. coli BL21 (0.5 mM IPTG, 20°C, 20 h) were purified using Ni-NTA agarose (Qiagen #30210). Cleared lysates were incubated with 50 μL beads (4°C, 4 h), washed with lysis buffer containing 20 mM imidazole, and eluted with 250 mM imidazole.

**5.16 Glucose Uptake, Pyruvate, Lactate, ATP, HK, PFK, ALDO, PK, and LDH Assays**

For the assessment of pyruvate and ATP levels, as well as PK activity, cells (5 × 10^5) were harvested and extracted using the specific assay buffers provided in the respective kits. The cells were centrifuged, and the supernatant was then measured spectrophotometrically at 570 nm using an enzymograph. For the determination of HK and ALDO activities, cells (5 × 10^5) were collected and homogenized in the appropriate assay buffers. The cells were centrifuged, and the supernatant was evaluated at 450 nm using an enzyme labeler. For the PFK activity assay, cells (2 × 10^6) were harvested and extracted with the PFK activity assay buffer. The cells were centrifuged, and the absorbance of the supernatant was measured at 450 nm using an enzymograph. For the LDH activity assay, cells (2 × 10^5) were harvested and homogenized in the LDH assay buffer. The cells were centrifuged, and the absorbance of the supernatant was measured at 450 nm using an enzymograph.

For lactate level determination, 10,000 cells were seeded into a 12-well plate and incubated in DMEM supplemented with 10% FBS for 10 hours. To assess lactate secretion, the medium was replaced with DMEM without FBS. After a 1-hour incubation, the supernatant was collected, and the lactic acid concentration was measured at 450 nm using an enzyme labeler. For intracellular lactate measurement, cell lysates were prepared from cultured cells or mouse retinal tissues. Tissues were homogenized in assay buffers, centrifuged, and the supernatant was analyzed and normalized to total protein concentration.

**5.17 Immunohistochemistry (IHC)**Paraffin-embedded retinas were deparaffinized in xylene, rehydrated through graded ethanol, and subjected to antigen retrieval (10 mM citrate buffer, pH 6.0, 95°C, 20 min). Endogenous peroxidases were quenched with 3% H₂O₂ (RT, 20 min). After blocking with 10% goat serum (RT, 1 h), sections were incubated with primary antibodies (RT, 1 h) followed by HRP-conjugated secondary antibodies (RT, 30 min). DAB substrate (Vector #SK-4100) was applied for 2 min, counterstained with hematoxylin (30 s), and imaged with a Nikon Eclipse 80i microscope.

**5.18 Chromatin Immunoprecipitation (ChIP)**RPE cells (5×10⁶) were crosslinked with 1% formaldehyde (10 min), quenched with 125 mM glycine, and sonicated to obtain 200-500 bp chromatin fragments. Chromatin was immunoprecipitated with 5 μg anti-H3K18la antibody (Jingjie, PTM-1406RM) or IgG control (CST #3900) using the EZ-ChIP kit (Millipore #17-10085). Precipitated DNA was analyzed by qPCR (primers in Table S2) with results expressed as % input = 2^(Ct_input - Ct_IP) ×100.

**5.19 RNA Fluorescence In Situ Hybridization (FISH)**RNA FISH was performed using the Beyotime kit (#R0306M) with optimized protocols. ARPE-19 cells grown on glass coverslips were fixed with 4% PFA (Beyotime #P0099, RT, 10 min), permeabilized with 0.5% Triton X-100/PBS (5 min), and hybridized with Cy5-labeled TXNIP probes (50 nM, denaturation at 65°C for 5 min) in hybridization buffer (37°C, 12 h dark). Post-hybridization washes included: 2× SSC/0.1% Tween-20 (42°C, 20 min), 1× SSC (37°C, 10 min), and 0.5× SSC (RT, 5 min). Nuclei were counterstained with DAPI (1 μg/mL, 5 min) and mounted with ProLong Diamond Antifade (Thermo #P36961). Imaging was performed on a Leica TCS SP8 STED microscope (63× oil, z-stack 0.3 μm).

**5.20 PLA**

Following the manufacturer's guidelines, the Duolink in situ test kit (Sigma, catalog number DUO92008) was utilized for proximity ligation assay (PLA) to determine adjacent connections. Briefly, cells seeded on a glass coverslip were fixed, permeabilized, blocked, and incubated with the designated primary antibody. Subsequently, the cells were hybridized with a PLA probe. Once the PLA signal was ligated and amplified, the PLA dots were captured through photography.

**Table S1: Targeted sequences of siRNAs and shRNAs**

**Gene names Targeted sequences**

si TXNIP #1 GGAGGUGUGUGAAGUUACUTT

si TXNIP#2 GUCAGAGGCAAUCAUAUUATT

sh *TXNIP* #1 GCAAGTAGCATCTCTGATT

sh *TXNIP* #2 GGTTTGGAATTGGCCGCTT

si *caspase-3* #1 GGATCGTTGTAGAAGTCTA

si *caspase-3* #2 GACGCTACTTTTCATGCAA

si *MLKL* #1 CTGGAGATATCCCGTTTCA

si *MLKL* #2 CGCTGTTACTTCAGGTTGA

si *GSDME* #1 CCCACTGCTTCTTTGTATA

si *GSDME* #2 CAAGCAGCTGTTTATGACA

**Table S2: Sequence of primers used in PCR analysis**

Name Forward primer (5’-3’) Reverse primer (5’-3’)

β-Actin CATGTACGTTGCTATCCAGGC CTCCTTAATGTCACGCACGAT

TXNIP CATCAGTCAGAGGCAATCA CCAGGAACGCTAACATAGAT

ERG AACGAGCGCAGAGTTATCG GTGAGCCTCTGGAAGTCGTC

EP300 AGCCAAGCGGCCTAAACTC TCACCACCATTGGTTAGTCCC
